# Meta-Analysis of Studies Using Suppression Subtractive Hybridization and Microarrays to Investigate the Effects of Environmental Stress on Gene Transcription in Oysters

**DOI:** 10.1371/journal.pone.0118839

**Published:** 2015-03-13

**Authors:** Kelli Anderson, Daisy A. Taylor, Emma L. Thompson, Aroon R. Melwani, Sham V. Nair, David A. Raftos

**Affiliations:** 1 Sydney Institute of Marine Science, Chowder Bay, NSW, Australia; 2 Department of Biological Sciences, Macquarie University, North Ryde, NSW, Australia; Glasgow Caledonian University, UNITED KINGDOM

## Abstract

Many microarray and suppression subtractive hybridization (SSH) studies have analyzed the effects of environmental stress on gene transcription in marine species. However, there have been no unifying analyses of these data to identify common stress response pathways. To address this shortfall, we conducted a meta-analysis of 14 studies that investigated the effects of different environmental stressors on gene expression in oysters. The stressors tested included chemical contamination, hypoxia and infection, as well as extremes of temperature, pH and turbidity. We found that the expression of over 400 genes in a range of oyster species changed significantly after exposure to environmental stress. A repeating pattern was evident in these transcriptional responses, regardless of the type of stress applied. Many of the genes that responded to environmental stress encoded proteins involved in translation and protein processing (including molecular chaperones), the mitochondrial electron transport chain, anti-oxidant activity and the cytoskeleton. In light of these findings, we put forward a consensus model of sub-cellular stress responses in oysters.

## Introduction

Human activity has introduced a range of stressors into coastal marine environments around the world. These stressors exacerbate natural variability and ramify throughout all levels of biological organization, from gene expression to the broader biosphere [1]. The main sources of environmental stress in coastal areas include chemical contamination, lack of food, altered hydrological parameters (temperature, pH, dissolved oxygen, turbidity etc.) and disease. Sewage, industrial activity, agricultural runoffs and storm water outfalls represent major sources of coastal pollution. They introduce organic and inorganic chemicals, such as eutrophying nutrients, toxic metals, polycyclic aromatic hydrocarbons (PAH), pesticides and polychlorinated biphenyls, into coastal waterways [2–5]. Hydrological changes resulting from coastal engineering can exacerbate natural fluctuations in water quality, particularly by increasing the frequency of hypoxic conditions during summer months and by changing localized water temperature, pH and dissolved CO_2_ concentrations [5].

Numerous studies have investigated the impacts of environmental stress on gene expression in coastal marine species (e.g., [6–9]). These studies have defined a range of genes that are affected by specific types of stress. However, the data have not been integrated to test whether there is a universal environmental stress response that is common to a broad range of different stressors. Understanding whether there is such a generic reaction to stress at the sub-cellular level will be critical for the development of management practices that can withstand multifaceted anthropogenic changes to the environment.

The current review addresses this deficit by undertaking a meta-analysis of transcriptome data to identify the key molecular systems that are affected by environmental stress in edible oysters. Oysters (phylum Mollusca, class Bivalvia, order Ostreoida, family Ostreidae) were chosen for this study because of their significance in global aquaculture, their role in significantly modifying habitats (ecosystem engineers), and their frequent use in environmental biomonitoring [10,11]. Oyster farming often relies on monocultures in impacted coastal environments. Hence, the detrimental effects of environmental stress are likely to be felt severely in these aquaculture industries [12]. The potential economic impacts of environmental change on oyster farming worldwide also means that substantial effort has been put into analyzing the effects of stress on oysters.

### Data mining and analysis

**Fig 1:**
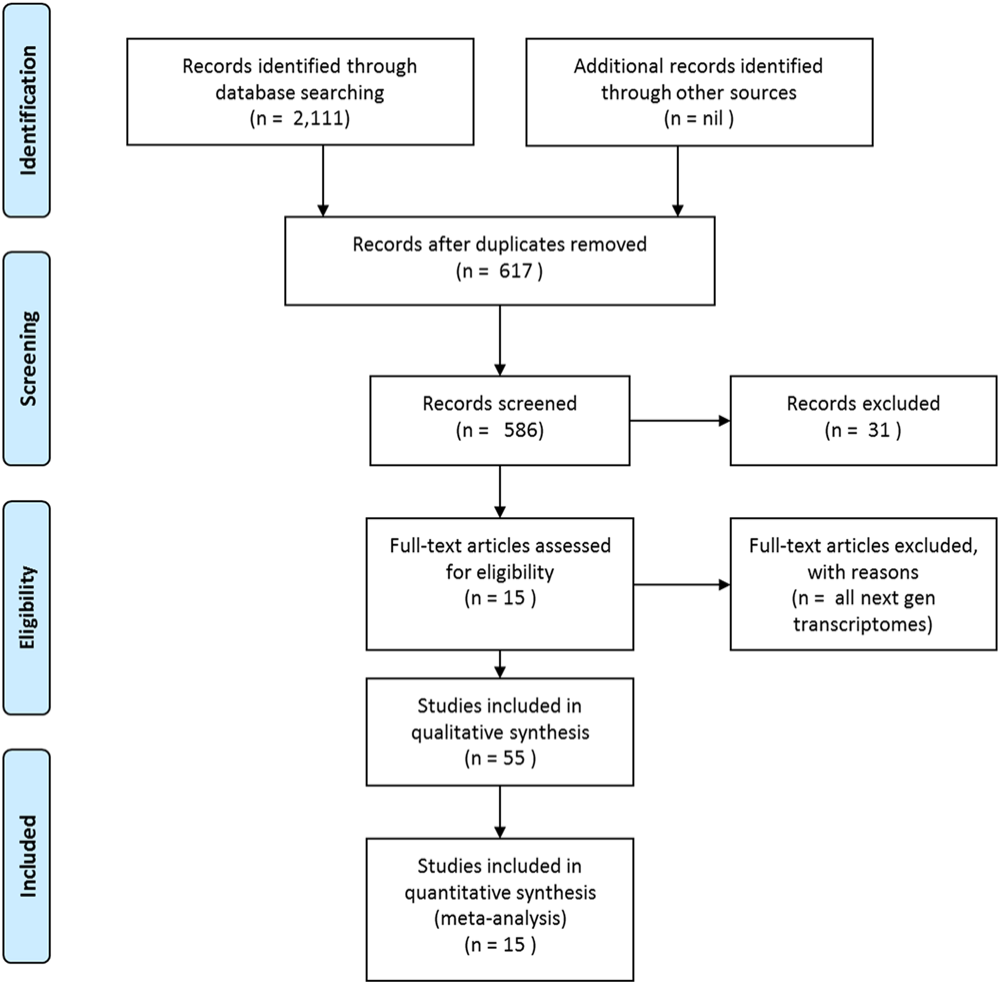
Schematic representation of the workflow used in this study.

Data for inclusion in the meta-analysis were selected by searching GenBank’s nucleotide and expressed sequence tag (EST) databases (http://www.ncbi.nlm.nih.gov) (Fig. 1). These databases were searched in 2013 for differentially expressed genes identified by SSH or cDNA microarray analyses of oysters from the genera *Crassostrea*, *Ostrea* and *Saccostrea* that had been exposed to environmental stressors (as defined by their GenBank annotations). Only microarray analyses that had been effectively validated by quantitative PCR were included in the analysis. Pertinent next generation transcriptomic and shotgun proteomic studies are discussed separately.

Once the full set of differentially expressed sequences with their associated accession numbers had been identified, they were assembled into a single database and re-analyzed to confirm their identity. In all of the following analyses, we used a cut-off ≤ e^-20^ to represent “robust” homology. All hits that did not meet this threshold were discarded. First, accession numbers associated with the differentially expressed GenBank entries were searched using Blast*n* for “somewhat similar sequences” against the non-redundant “other” nucleotide database on NCBI. Robust hits (hits against homologous genes with ≤ e^-20^ matches) were included in our raw database. All genes that failed to meet this criterion using Blast*n* were searched again using Blast*x* (“translated nucleotide query against protein database”) with the default settings. Genes identified using Blast*x* searches with ≤ e^-20^ were also included in the raw database.

When the top blast hits in Blast*n* or Blast*x* searches had gene names that were different to that assigned to the original oyster sequence, both names were searched via Uniprot (http://www.uniprot.org/) to determine whether they were synonyms. For instance, NADH dehydrogenase (EC 1.6.99.3) was deemed to be synonymous with cytochrome c reductase, NADH oxidoreductase and 13 other gene names; see http://enzyme.expasy.org/EC/1.6.99.3). In many cases, the alternative gene name was added to our database annotation to avoid redundancy. In addition, if two or more of the entries in our database of oyster genes encoded the same gene but had been allocated different names in the literature, those redundant sequences were consolidated into a single entry. In some additional cases, genes encoded in the mitochondrial (Mt) genome were allocated accession numbers in the primary literature that corresponded to the entire Mt genome. These were re-allocated gene-specific accessions.

After this filtering process, the final consolidated database was re-analyzed to verify the putative functions of the differentially expressed genes. These functional assignments were initially checked against searches of the literature cited in the GenBank registration file for each sequence. The functional category into which the gene fell was then confirmed by reference to the UniProtKB Protein Knowledgebase (http://www.uniprot.org/uniprot/) and the primary literature. The results from these searches were cross-referenced and hierarchically preferenced so that a single biological function could be assigned to each gene sequence in the database. Preference was given to ontologies that were based on experimentally defined functions in oysters, other bivalves, and mollusks in general.

As a result of these multiple filters, only sequences from published studies with definitive gene identifications and robust functional annotations were included in subsequent analyses.

At the time of this analysis, there were 97,811 nucleotide and 223,784 EST sequences listed in GenBank under the three oyster genera investigated (Table 1). Ninety-eight percent of the CoreNucleotide sequences that we searched against were from the genus *Crassostrea*, 0.6% were from *Saccostrea*, and 1.5% were from *Ostrea*. More than 99% of the dbEST sequences were from the genus *Crassostrea*. These relative numbers of transcriptome sequences match closely with worldwide aquaculture production levels for the three genera. Over 99% of the edible oysters produced globally in 2009 were from the genus *Crassostrea* (primarily *C*. *gigas*; http://www.fao.org/). This demonstrates that, in the case of oysters, the depth of available sequence resources matches the worldwide economic importance of particular species.

**Table 1:** Total numbers of nucleotide and EST sequences for the oyster genera *Crassostrea*, *Saccostrea* and *Ostrea* in GenBank via NCBI (as at July, 2013) that candidate sequences were searched against in the current analysis.

Genus	CoreNucleotide	dbEST
*Crassostrea*	95,743	223,547
*Saccostrea*	569	228
*Ostrea*	1,499	9
Total	97,811	223,784

### Structure of the differentially expressed gene database

2,111 of the transcript sequences in GenBank databases were annotated as being differentially expressed in oysters that had been exposed to environmental stress. The filtering process described above, which checked the identity and putative functions of the differentially expressed genes, compacted the initial list of 2,111 sequences into a consolidated database of 617 validated non-redundant genes. The final edited list of these genes is shown in S1 Table. When genes with no known function and those not assigned a functional category (as well as equivocal entries) were removed, this list was reduced to 586 known genes with predicted functions. The differentially expressed transcripts were identified in 14 studies that used cDNA microarrays (four studies) or SSH (10 studies) to test the effects of environmental stress on oysters (Table 2). Six of the 14 studies tested gene expression in the hemolymph of oysters, four used digestive gland tissue, and the remainder used a combination of gills, mantle and/or gonads as the source of mRNA. The environmental stressors to which oysters were exposed included, hydrocarbons and other organic contaminants, metals, infectious agents, pesticides, hypoxia, extremes of pH, and thermal stress.

**Table 2:** Studies used in this meta-analysis.

*Stressor*	*Species*	*Technology*	*Target tissue*	*Reference*
**Hydrocarbon**	*C*. *gigas*	SSH	Digestive gland	Boutet et al. [6]
**Metals/organic contaminants/water quality parameters**	*C*. *virginica*	MA	Gill, hepatopancreas	Chapman et al. [7]
**Hypoxia**	*C*. *gigas*	SSH	Digestive gland, gill-mantle	David et al. [13]
**QX (Queensland Unknown) disease**	*S*. *glomerata*	SSH	Hemolymph	Green et al. [42]
**Bacteria**	*C*. *gigas*	MA	Hemolymph	Gueguen et al. [8]
***Vibrio splendidus***	*C*. *gigas*	SSH	Mantle, gonad	Huvet et al. [14]
***Bonamia ostrea***	*O*. *edulis*	SSH	Hemolymph	Martín-Gómez et al. [15]
**Thermal stress**	*C*. *gigas*	SSH	Gill, mantle	Meistertzheim et al. [9]
***Bonamia ostrea***	*O*. *edulis*	SSH	Hemolymph	Morga et al. [16]
**Ostreid herpesvirus 1**	*C*. *gigas*	SSH	Hemolymph	Renault et al. [17]
**Hypoxia**	*C*. *gigas*	MA	Hemolymph	Sussarellu et al. [18]
***Perkinsus marinus***	*C*. *gigas*, *C*. *virginica*	SSH	Hemolymph	Tanguy et al. [19]
**Glyphoshate/pesticides**	*C*. *gigas*	SSH	Digestive gland	Tanguy et al. [20]
***Perkinsus marinus***	*C*. *virginica*	MA	Gill	Wang et al. [21]

In this list, “metals” denotes arsenic, cadmium, copper, chromium, iron, mercury or lead; “organic contaminants” were chloropyrifos, naphthalene, DDT and polycyclic aromatic hydrocarbons (PAHs); and “water quality parameters” were extremes of temperature, salinity, pH, turbidity, dissolved oxygen, chlorophyll a, and ammonia. Abbreviations: MA, microarray; SSH, suppression subtractive hybridization.

Six of the 14 studies included in the meta-analysis provided consistent data on the up- or down- regulation of differentially expressed genes [6, 13, 15, 18, 20, 21]. Of the 1352 differential genes identified in these studies, 59% ± 18% were upregulated in response to stress. However, a further time course analysis [9], showed that individual genes could either be up- or down- regulated depending on the duration of exposure to stress.

### Ontology of differentially expressed genes

The functional ontologies of the mined genes showed that 12 discrete intracellular processes were affected by ‘stress’. The abbreviated titles for these 12 functional categories are shown in Table 3. The percentages cited in the following discussion represent the percentage of the total differential transcriptome (586 genes) comprising a particular functional category. Eight percent of the differentially expressed genes had no known function and thus could not be assigned to a functional category. Only 1% of the differentially expressed genes fell into functional categories other than the 12 listed in Table 3.

**Table 3:** Abbreviations assigned to the twelve functional gene categories that were affected by environmental stress.

Intracellular process	Designated sub-categories	Abbreviation
Cell differentiation, proliferation and apoptosis (includes cell cycle regulation)	Cell death	*Cell cycle*
Cell communication, membrane receptors, intracellular signalling		*Communication*
Cytoskeleton, cell structure and vesicular trafficking		*Cytoskeleton*
Extracellular matrix		*Extracellular matrix*
Immunity (and wound healing)		*Immunity*
Metabolism (includes lipid binding and lipid metabolism)	Electron transport, Glycolysis and TCA cycle	*Metabolism*
Nucleic acid regulation (includes DNA repair)		*Nucleic acid regulation*
Protein regulation	Proteases, Proteosomes	*Protein regulation*
Trans-membrane gates, channels and pumps		*Membrane transport*
Detoxification and intracellular stress (includes molecular chaperones)	Chaperones, Oxidative stress	*Stress*
Transcription (includes transcriptional regulation)	Nucleosomes	*Transcription*
Translational and post-translational processing (includes protein trafficking)	Ribosomes	*Translation*
Other intracellular processes		*Other*
No ascribed function		*Unknown*

**Fig 2:**
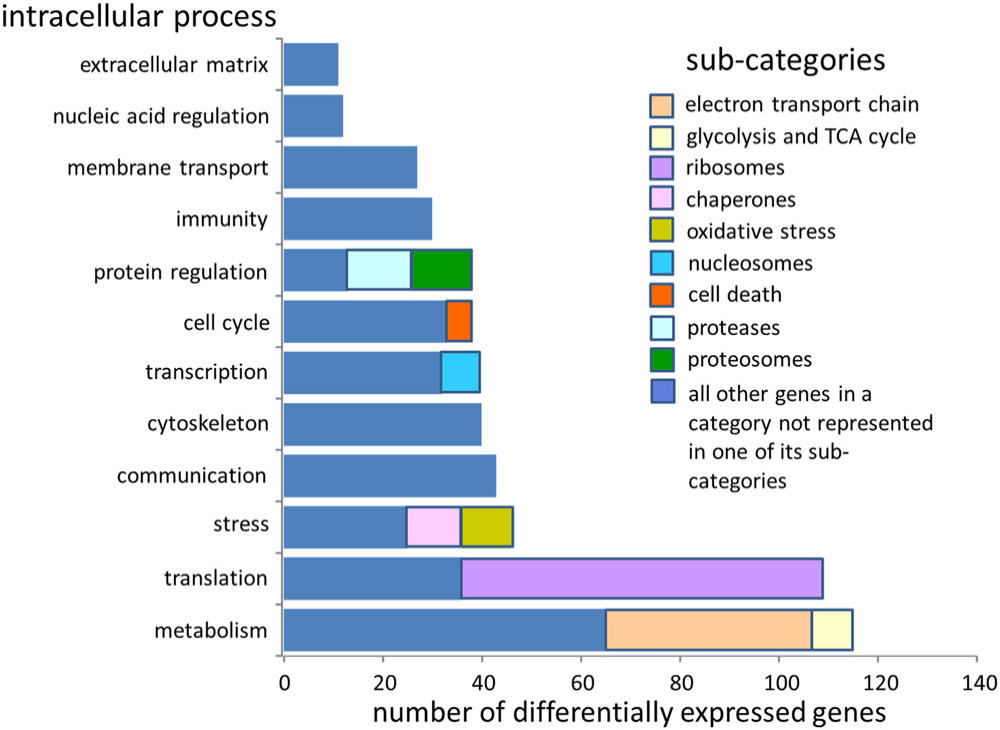
The number of differentially expressed genes from all environmental treatments that fell into discrete intracellular process categories.

Genes associated with metabolism were the most frequently identified (Fig. 2; 28%). These included genes involved in the mitochondiral electron transport chain (10%) and in glycolysis and the tricarboxylic acid (TCA/Krebs) cycle (2%). Genes associated with translation and post translational processing were the next most abundant category, comprising 26% of the differential transcriptome. Most of these genes encoded ribosomal proteins or rRNA (18%). Stress response genes, including molecular chaperones (2%) and enzymes involved in antioxidant responses (including superoxide dismutase, SOD, and peroxiredoxin, 2%), accounted for 11% of differential transcripts. Communication, the cytoskeleton, transcription, the cell cycle and protein regulation each represented approximately 10% of the differential transcriptome. Genes involved in immune responses and membrane transport each accounted for approximately 7% of differentially expressed transcripts. Nucleic acid regulation and the extracellular matrix were the least represented functional catgories (3% or less of the differential transcriptome). The most frequently identified genes in each functional group are shown in Table 4.

**Table 4:** The top five most commonly identified types of differentially expressed genes in the main intracellular process categories affected by environmental stress.

Intracellular process	Top five most commonly identified types of genes in ranked order (number of times identified)				
Cell cycle	DNA topoisomerases (4)	Apoptosis factors (4)	Cyclins (3)		
Communication	G proteins (18)	Rho-like proteins (GTPases) (7)	Calmodulins (6)	Importins (2)	Integrins (2)
Cytoskeleton	Actins (12)	Tubulins (12)	Profilins (3)	Dyenins (3)	Myosins (3)
Extracellular matrix	Hemicentins (3)	Tenascins (3)	Collagens (3)	Fibrillins (2)	
Immunity	Lectins (15)	LPS/β-glucan binding proteins (7)	Complement components (4)	Ferritin (2)	Defensins (2)
Metabolism	NADH dehydrogenases (29)	Cytochrome c oxidases (18)	ATP synthases (16)	Acyl-CoA associated proteins (16)	Fatty acid binding proteins (5)
Nucleic acid regulation	Ribonucleotide reductases (2)				
Protein regulation	Ubiquitins and associated proteins (12)	Cathepsins (7)	α2-macroglobulin-like proteins (4)	TIMPs (2)	Cystatins (2)
Membrane transport	Ion channels (9)	Solute carrier family members (8)	Amino acid transporters (6)	Glucose transporters (3)	Lysosomal membrane proteins (2)
Stress	Superoxide dismutases (8)	Metallothioneins (7)	Glutathione-S-transferases (6)	Heat shock protein 70's (4)	Glutathione peroxidases (4)

The number of times a gene, sub-units of the protein encoded by that gene, homologues of the gene, or associated gene products were identified in different studies of environmental stress is shown in brackets. Only genes that appeared more than once in the dataset are shown.

**Fig 3:**
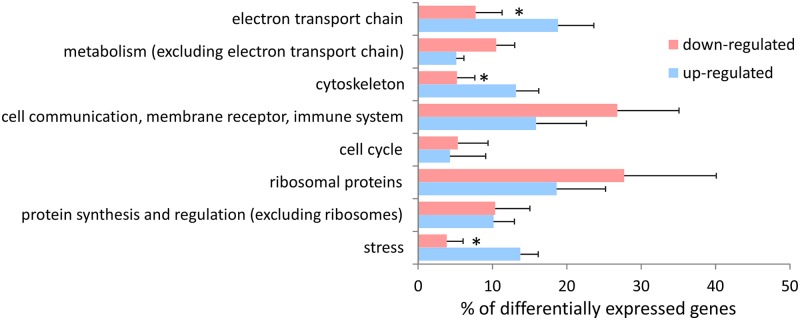
The percentage of genes within discrete intracellular process categories that were reported as either up- or down-regulated by the original authors [6, 13, 15, 18, 20, 21]. Bars = SEM (n = 6 different studies that verifiably reported direction of change), * denotes a significant difference between up- and down-regulation for that category (t-test, p < 0.05).

The direction of change (up- or down- regulation) of genes comprising the different functional categories is shown in Fig. 3 (using data from [6, 13, 15, 18, 20, 21]). The proportion of genes that were up-regulated, relative to down-regulation, was significantly greater (t-test, p < 0.05) in the cellular stress response, cytoskeleton and electron transport chain categories.

### The individual genes most frequently affected by stress are involved in the mitochondrial electron transport chain, translation and protein processing, subcellular stress responses and the cytoskeleton

**Fig 4:**
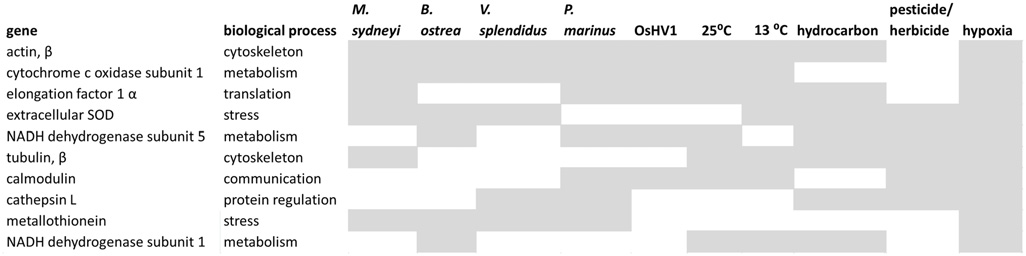
The top ten genes whose expression was affected by the greatest number of environmental stressors. Genes are listed in ranked order from most frequently affected to least frequently affected. Shaded boxes represent significantly altered gene expression in response to different stressors.

None of the 586 individual genes identified in this analysis was found to be differentially expressed in all 10 of the stress treatments analysed (5 different types of infectious agents, two temperature extremes, two types of contaminants, and hypoxia) (Fig. 4). Actin β was the most frequently affected gene. It was differentially expressed in all treatments except “pesticide/herbicide”. Cytochrome oxidase subunit 1 (CO1) expression was effected by eight out of the 10 stressors. Commonly affected genes such as actin and CO1 were relatively rare. Only 4% of the 586 genes in this analysis were differentially expressed in more than 6 of the treatments, and the expression of 60% of genes was affected by just one of the 10 stress treatments.

**Fig 5:**
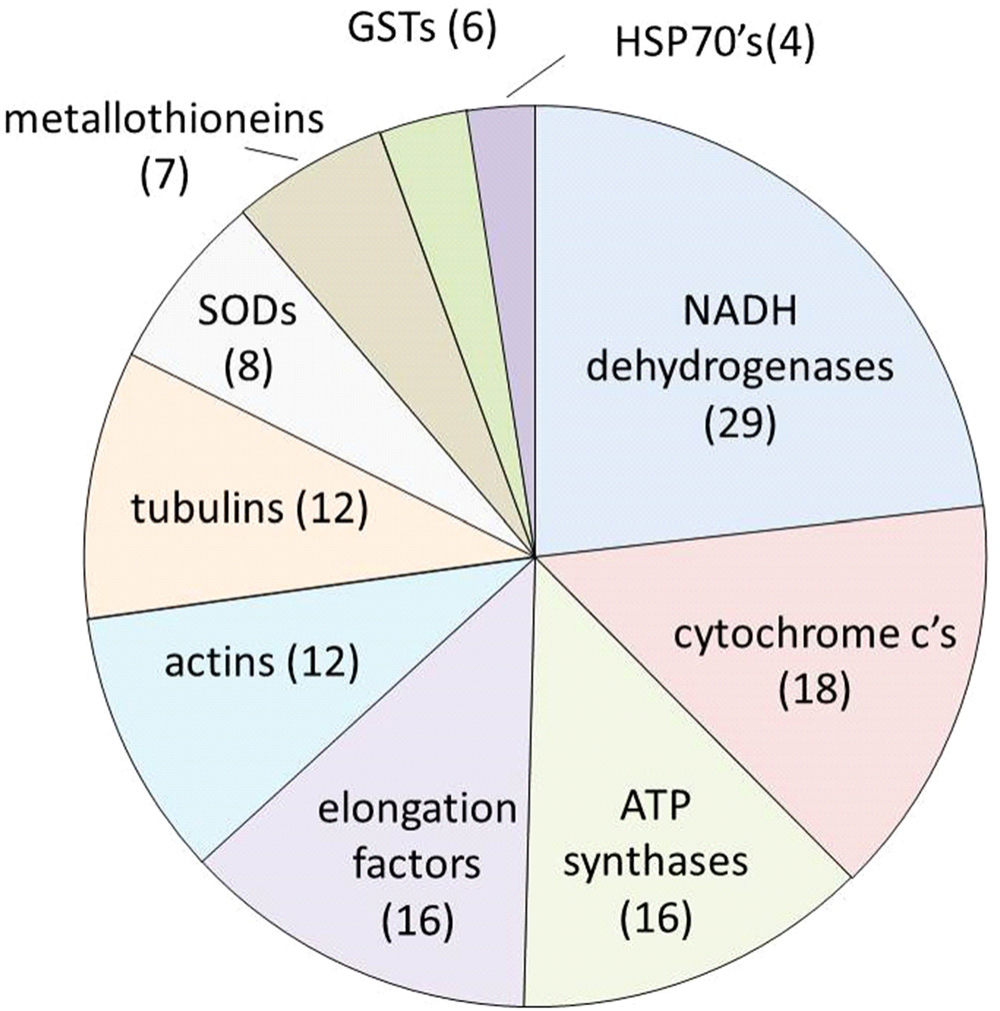
The ten most common differentially expressed genes identified by “name” (comprising more than one accession number) in oysters exposed to 10 different types of environmental stressors. Numbers in brackets represent the number of times this gene name appeared as differentially expressed in all of the different stress treatments. These data do not include genes encoding ribosomal proteins.

Fig. 5 shows the 10 most common differentially expressed genes (excluding ribosomes) across all stress treatments identified by name rather than accession number. These “names” represented groups of genes that encoded either different polypeptides of the same protein (for instance NADH dehydrogenase subunits), functionally related homologues in the same gene family (for example HSP70s), or synonyms for the same protein (for instance NADH dehydrogenase and NADH oxidoreductase). This pooling meant that the same “name” could occur more than once in the same stress treatment. Together, the top ten “names” accounted for 31% of the 586 differentially expressed genes identified across the 10 stress treatments. NADH dehydrogenases were by far the most commonly identified differential transcripts. By themselves, they accounted for 7% of the differential transcriptome. Their transcript abundance was significantly affected by all stressors. Among the top 10 gene names, those associated with the mitochondrial electron transport chain (NADH dehydrogenases, cytochrome c’s and ATP synthases) represented 16% of the entire differential transcriptome, whilst cytoskeletal (actin and tubulin) and stress response (metallothioneins, GSTs, HSP70s, and the antioxidant enzyme SOD) genes each comprised 6% of all differential transcripts.

### Effects of different types of stress on gene expression

Too few differentially expressed genes were identified in some of the 10 different stress treatments to allow robust statistical comparisons between all treatments. So, to allow us to compare the effects of different types of stressors, we collapsed the analysis to compare just 4 different general classes of stressor (infection, temperature, contamination and hypoxia). We also eliminated the least affected functional categories of genes (extracellular matrix, nucleic acid regulation and membrane transport), and combined transcription and translation into a single functional category.

**Fig 6:**
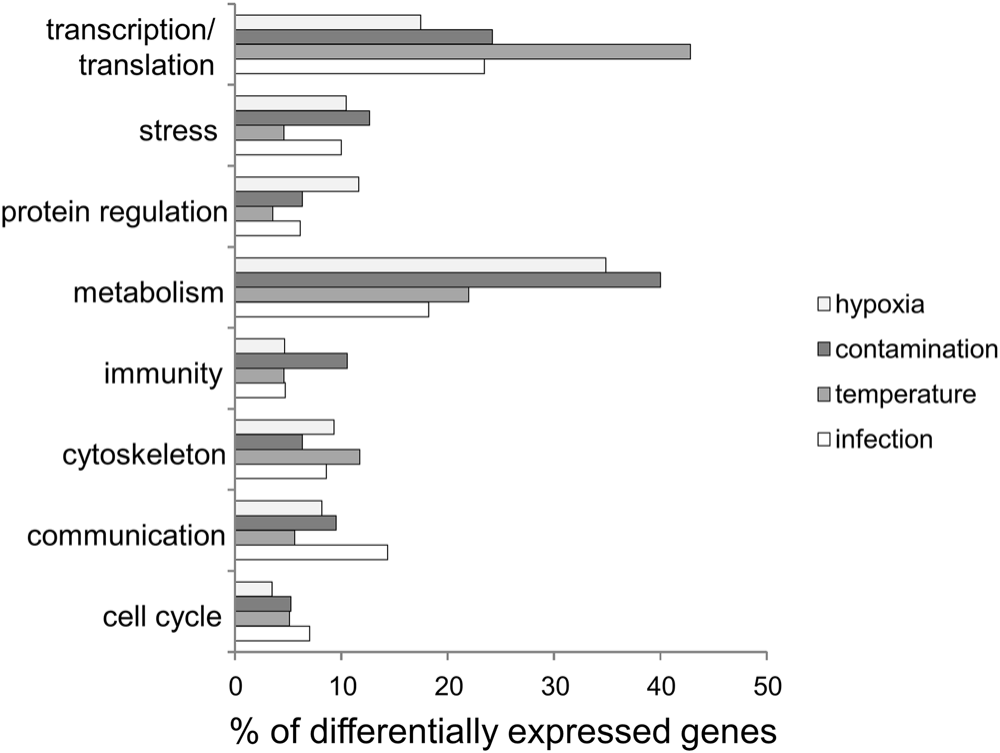
Biological functions of genes that were differentially expressed in response to four general categories of stress: hypoxia (n = 101 genes); contamination (hydrocarbons, metals, and pesticides, n = 115); temperature extremes (high and low, n = 280); and, infection (*Bonamia ostrea, Martelia sydneyi, Perkinsus marinus*, generic bacterial sp., *Vibrio splendidus*, and *Ostreid herpes virus 1*, n = 705).

The percentage of differentially expressed genes comprising the different functional categories was broadly similar between all four general classes of stressors (Fig. 6). Genes in the transcription/translation and metabolism categories were the most frequently affected by all classes of stress. However, there was significant variability (χ^2^ = 73.23, df = 21, p < 0.05) in the responses to different stressors. Temperature extremes affected the expression of significantly more genes involved in transcription and/or translation than the other types of stress. Similarly, hypoxia and contamination had a proportionally greater effect on genes involved in metabolism when compared to temperature extremes and infection. Despite these minor variations, the transcriptional profiles of oysters responding to the different stressors were quite similar. This suggests that there is a core set of stress response genes within oyster cells, primarily involved in metabolism, and that the transcription/translation of these genes is responsive to a broad range of environmental stressors. By corollary, the data imply that enhanced cellular energy demand is a universal response to environmental stress, and that adaptive responses to stress extend well beyond the “minimal stress proteome of cellular organisms” proposed by Kültz [22].

### Genomic, next generation transcriptomic and proteomic analyses of environmental stress in oysters

A complete genome sequence is now available for Pacific oysters (*C*. *gigas*) [23], and “omics” data for edible oysters continues to expand [24]. The *C*. *gigas* genome project revealed substantial expansion, relative to other species, of gene families such as heat shock protein 70s that are involved in environmental stress responses. This was deemed to be an adaptive response to the highly stressful intertidal habitats occupied by *C*. *gigas* [23]. Other recent studies have used next generation analytical techniques to investigate stress responses in oysters; including RNAseq transcriptomic responses to salinity stress [25], virulent Vibrio strains [26] and Roseovarius Oyster Disease (ROD) [27], and shotgun proteomic analyses of heavy metal contamination [28] and elevated *p*CO_2_ [29]. These studies found differential responses to environmental stress in many of the same subcellular pathways identified in the current meta-analysis. For instance, Timmins-Schiffman et al. [29] showed that changes in the proteomes of *C*. *gigas* responding to elevated *p*CO_2_ included pathways involved in antioxidant activity, carbohydrate metabolism and transcription/ translation. Similarly, a shotgun proteomic analysis by Muralidharan et al. [28] found that proteins encoded by six of the ten most commonly affected genes identified in the current meta-analysis (Fig. 4) were also differentially regulated in *S*. *glomerata* after exposure to heavy metals. The differential proteins included NADH dehydrogenase, cytochrome c oxidase, tubulin, actin and ATP synthase. These data suggest that next generation studies investigating stress responses in oysters tend to identify the same intracellular stress response pathways as those revealed by SSH and microarrays. Based on the relatively few existing next generation analyses, these newer techniques do not seem likely to discover entirely novel mechanisms. However, their greater analytical depth will no doubt detect more components of pathways already identified by previous studies, and they may be able to discern subtle differences in responses to different types of stress. This might be particularly useful if the goal of analyses is to identify suites of biomarkers for environmental monitoring.

### Conclusions and perspectives

**Fig 7:**
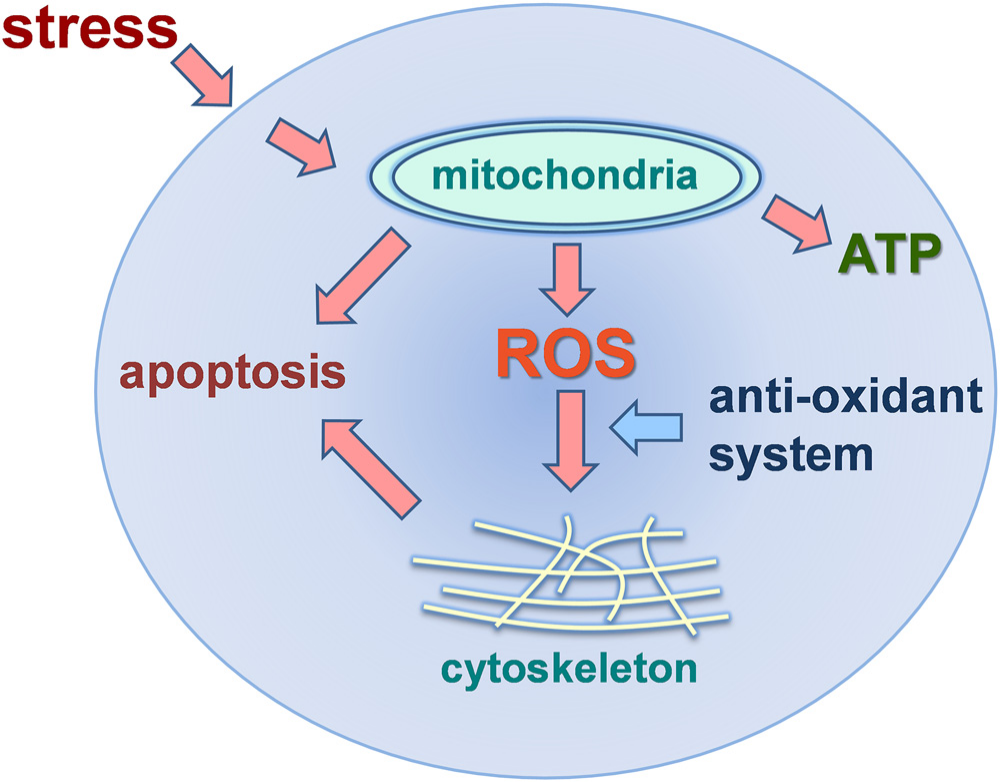
A consensus model of intracellular responses to stress in oysters based on the genes and/or biological processes that were found in our analysis to be differentially regulated in response to environmental stress. In this model, adaptive intracellular responses to stress lead to increased energy production (ATP) via the mitochondrial electron transport chain. Reactive oxygen species (ROS) are produced as by-products of ATP synthesis. Unless controlled by molecular chaperones (HSPs) and the anti-oxidant system, excessive ROS can disrupt the actin cytoskeleton and mitochondrial membranes leading to apoptotic cell death.

This meta-analysis has compiled data from 14 different transcriptomic studies of oysters exposed to a range of environmental conditions. It has shown that a number of key sub-cellular systems are consistently involved in transcriptional responses to a broad range of environmental stresses. The data support a consensus model that describes a generic intracellular stress response in oysters (Fig. 7). Elements of this model, as it pertains to oysters and other organisms, have been discussed in detail by other authors [30–31]. The model highlights the importance of the mitochondrial electron transport chain, antioxidant enzymes, molecular chaperones and cytoskeletal proteins. Our meta-analysis has shown that the mitochondrial electron transport chain is the primary cellular system impacted by environmental stress and so it is central to the consensus model. Even though enhanced energy production in the mitochondria may be beneficial in terms of powering adaptive cellular processes, it is also known to elevate the production of cytotoxic reactive oxygen species (ROS), with consequent impacts on mitochondrial and cytoskeletal integrity. The failure of anti-oxidant enzymes such as SOD and molecular chaperones to limit damage caused by ROS is likely to result in cellular dysfunction and ultimately apoptotic cell death. All of these effects have been demonstrated in numerous studies of cellular function in oysters responding to stress [32–49]. Modulation of the mitochondrial electron transport chain and its downstream consequences, as depicted in this consensus model, may explain many of the impacts that environmental stress exerts at levels of biological organization from cells to ecosystems [32].

## Supporting Information

S1 PRISMA Checklist PRISMA checklist. (DOC)

S1 Table The complete list of differentially expressed genes affected by environmental stress identified in this meta-analysis showing; the gene or encoded protein name, the intracellular process to which the gene was assigned, the accession number for the gene and the type of stress that significantly affected the expression of the gene (designated by X in the corresponding box). Abbreviations: Comms, Communication; QX, QX disease/*Martelia sydneyi* infection, B. ost, *Bonamia ostrea*; V. spl, *Vibrio splendidus*; HV1. Ostreid Herpesvirus 1; P. mar, *Perkinsus marinus*; temp high, high temperature; temp low, low temperature; HC, hydorcarbons; pest/herb, pesticides/herbicides; hypox, hypoxia.
(PDF)
