# Supplementary material for: Meta-Analysis of Studies Using Suppression Subtractive Hybridization and Microarrays to Investigate the Effects of Environmental Stress on Gene Transcription in Oysters
Source: PLoS One. 2015 Mar 13;10(3):e0118839. doi: 10.1371/journal.pone.0118839 (PMC4358831; doi:10.1371/journal.pone.0118839)
Supplement: S1 Table — Abbreviations: Comms, Communication; QX, QX disease/Martelia sydneyi infection, B. ost, Bonamia ostrea; V. spl, Vibrio splendidus; HV1. Ostreid Herpesvirus 1; P. mar, Perkinsus marinus; temp high, high temperature; temp low, low temperature; HC, hydorcarbons; pest/herb, pesticides/herbicides; hypox, hypoxia. (PDF) [file pone.0118839.s002.pdf]

**Table S1**

The complete list of differentially expressed genes affected by environmental stress identified in this meta-analysis showing; the gene or encoded protein name, the intracellular process to which the gene was assigned, the accession number for the gene and the type of stress that significantly affected the expression of the gene (designated by X in the corresponding box). Abbreviations: *Comms*, Communication; *QX*, QX disease/*Martelia sydneyi* infection, *B. ost*, *Bonamia ostrea*; *V. spl*, *Vibrio splendidus*; HV1. Ostreid Herpesvirus 1; *P. mar*, *Perkinsus marinus*; *temp high*, high temperature; *temp low*, low temperature; *HC*, hydorcarbons; *pest/herb*, pesticides/herbicides; *hypox*, hypoxia.

| **Gene/Protein** | **Intracellular Process** | **Accession #** | **QX** | ***B. ost*** | ***V. spl*** | **HV 1** | ***P. mar*** | **temp high** | **temp low** | **HC** | **pest/ herb** | **hypox** |
|---|---|---|---|---|---|---|---|---|---|---|---|---|
| 14kDa subunit splicing factor 3b | Translation | GH612367 | x | x | | | | | | | | |
| 16S ribosomal sequence, mitochondrial | Translation | JF744718 | | x | | | | | | | | |
| 18S ribosomal RNA | Translation | JF744719 | | x | | | | | | | | |
| 2,4-dienoyl CoA reductase 2 | Metabolism | CB617391 | | | | | | | | x | | |
| 40S ribosomal protein s12 | Translation | GH612387 | x | | | | | | | | | |
| 40S ribosomal protein s15a | Translation | CK172414 | | | x | | | | | | | |
| 40S ribosomal protein S16 | Translation | EE677841 | | | | | | | x | | | |
| 40S ribosomal protein S2 | Translation | EU108718 | | | | | | | x | | | |
| 40S ribosomal protein S23 | Translation | EE677844 | | | | | | x | | | | |
| 40S ribosomal protein S24 | Translation | EE677846 | | | | | | | x | | | |
| 40S ribosomal protein S26 | Translation | EE677848 | | | | | | | x | | | |
| 40S ribosomal protein S3-B (S1B) | Translation | EE677850 | | | | | | | x | | | |
| 40S ribosomal protein S6 | Translation | EE677852 | | | | | | | x | | | |

| Gene/Protein | Intracellular Process | Accession # | QX | *B. ost* | *V. spl* | HV 1 | *P. mar* | temp high | temp low | HC | pest/ herb | hypox |
|---|---|---|---|---|---|---|---|---|---|---|---|---|
| 40S ribosomal protein S9 | Translation | EE677856 | | | | | | x | | | | |
| 40S ribosomal protein SA | Translation | EE677858 | | | | | | x | | | | |
| 5' Methylthioadenosine phosphorylase | Metabolism | CD526712 | | | | | x | | | | | |
| 5' nucleotidase precursor | NAR | CB617398 | | | | | | | | x | | |
| 5-Aminolevulinate synthase | Metabolism | EU073062 | | | | | | | | | | |
| 60S ribosomal protein L11 | Translation | EE677862 | | | | | | | x | | | |
| 60S ribosomal protein L13A | Translation | EE677864 | | | | | | x | x | | x | x |
| 60S ribosomal protein L14 | Translation | CX069164 | | | | | | x | x | | | |
| 60S ribosomal protein L15 | Translation | DW713892 | | | | | | x | x | | | |
| 60S ribosomal protein L38 | Translation | EE677867 | | | | | | | x | | | |
| 60S Ribosomal protein L44 | Translation | GH612238 | x | | | | | | | | | |
| 60S ribosomal protein L6 | Translation | EE677869 | | | | | x | x | x | | x | |
| 60S ribosomal protein P0 (L10E) | Translation | EE677860 | | | | | | | x | | | |
| 60S ribosomal protein P2 | Translation | DW713852 | | | | | | x | x | | | |
| 67kD laminin receptor precursor | Comms | EE677695 | | | | | | x | | | | |
| Acetylcholinesterase/ butyrylcholinesterase/Cholinesterase | Comms | HS513818 | | | | x | | | | | | |
| Acetyltransferase, GNAT family | Transcription | EE677696 | | | | | | x | | | | |

| Gene/Protein | Intracellular Process | Accession # | QX | *B. ost* | *V. spl* | HV 1 | *P. mar* | temp high | temp low | HC | pest/ herb | hypox |
|---|---|---|---|---|---|---|---|---|---|---|---|---|
| Actin 5C | Cytoskeleton | CX069247 | | | | | | | | | | x |
| Actin cytoplasmic A3 | Cytoskeleton | CF369133 | | | | | x | | x | | x | |
| Actin depolymerising factor | Cytoskeleton | GH612316 | x | | | | | | x | | | x |
| Actin, β | Cytoskeleton | GH612256 | x | x | | x | x | x | x | x | | x |
| Acyl CoA oxidase similar protein | Metabolism | EU108716 | | | | | | | | | | |
| Acyl-CoA desaturase | Metabolism | HS513791 | | | | x | | x | | x | | x |
| Adaptor related complex (AP-3) | Cytoskeleton | CD526736 | | | | | x | | | | | |
| Adenosylhomocysteinase | NAR | CX069215 | | | | | | | | | | x |
| ADP-ribosylation factor 2 | Cytoskeleton | CF369249 | | | | | | | | | x | x |
| Adseverin-like protein | Cytoskeleton | JF744695 | | x | | x | | | | | | |
| Agglutination-aggregation factor 18K-LAF | Immunity | DW713832 | | | | | | x | | | | |
| Agrin | Comms | CX069319 | | | | | | | | | | x |
| Alcohol dehydrogenase class III chain | Metabolism | CX069325 | | | | | | | | | | x |
| Aldehyde dehydrogenase family 6, subfamily A1 | Metabolism | EE677697 | | | | | | | x | | | |
| Alkaline phosphatase | Metabolism | CB617556 | | | | | | | | x | | |
| Alpha macroglobulin | Protein regulation | HS513827 | | | | x | | | | | | |

| **Gene/Protein** | **Intracellular Process** | **Accession #** | **QX** | ***B. ost*** | ***V. spl*** | **HV 1** | ***P. mar*** | **temp high** | **temp low** | **HC** | **pest/ herb** | **hypox** |
|---|---|---|---|---|---|---|---|---|---|---|---|---|
| Alpha NAC | Translation | CD526829 | | | | | x | | | | | |
| Alpha/beta hydrolase | Metabolism | HS513808 | | | | x | x | | | | | |
| Alpha-2 adrenergic receptor | Comms | GH612269 | x | | | | | | | | | |
| alpha-2-macroglobulin, N-terminal and alpha-2-macroglobulin family member | Protein regulation | CX069254 | | | | | | | | | | x |
| Alpha-crystallin B chain | Unknown | EE677747 | x | | | | | x | | | | |
| Alternative oxidase isoform A | Metabolism | EU108720 | | | | | | | | | | |
| Amino acid transporter | Pumps | DW713848 | | | | | x | x | | x | | x |
| Amyloid protein A | ECM | DW713847 | | | | | | x | | | | |
| Ankyrin repeat domain 23 | cytoskeleton | JF744726 | | x | | | | | | | | |
| Anxa6 protein | Unknown | GH612414 | x | | | | | | | | | |
| Apextrin | Cell cycle | CX069241 | | | | | | | | | | x |
| Apolipophorin precursor | Metabolism | CB617501 | | | | | | | | x | x | |
| Archeron-like protein | Cell cycle | EU678313 | | | | x | | | | | | |
| Arginine kinase | Metabolism | CD646729 | | | | | x | | | | | x |
| arginine N-methyltransferase 5 | Metabolism | EE677771 | | | | | | | x | | | |
| Arginyl-tRNA synthetase | Translation | CB617392 | | | | | | | | x | | |
| Asparaginyl tRNA-synthetase | Metabolism | EU108715 | | | | | x | | | | | |

| Gene/Protein | Intracellular Process | Accession # | QX | *B. ost* | *V. spl* | HV 1 | *P. mar* | temp high | temp low | HC | pest/ herb | hypox |
|---|---|---|---|---|---|---|---|---|---|---|---|---|
| Aspartate amino transferase | Metabolism | AJ496218 | | | | | | | | x | | |
| ATP syntase alpha subunit | Metabolism | CF369142 | | | | | | x | | | x | x |
| ATP syntase beta subunit | Metabolism | CF369132 | | | | | | | x | | x | |
| ATP synthase | Metabolism | EE677738 | | | | | | x | | | | |
| ATP synthase F0 subunit 6 | Metabolism | EE677700 | x | x | | | | x | | | | |
| ATP synthase subunitB | Metabolism | GH612404 | x | | | | | | | x | | |
| Atpase H+transporting lysosomal protein | Pumps | CK172372 | | | x | | | | | | | |
| ATP-binding cassette transporter sub-family a member 3 | Pumps | EH645116 | | | | | x | | | | | |
| ATP-gated ion channel subunit P2X4 | Metabolism | CB617507 | | | | | | | | x | | |
| ATP-synthase subunit D | Metabolism | HS513814 | | | | x | | | | | | |
| Baculoviral IAP repeat-containing protein 2 | Cell cycle | EE677701 | | | | | | x | | | | |

| Gene/Protein | Intracellular Process | Accession # | QX | *B. ost* | *V. spl* | HV 1 | *P. mar* | temp high | temp low | HC | pest/ herb | hypox |
|---|---|---|---|---|---|---|---|---|---|---|---|---|
| Band 4.1-like protein 3 | Cell cycle | EE677719 | | | | | | x | | | | |
| Basic transcription factor 3-like 4 | Cell cycle | DW713855 | | | | | | x | x | | | |
| Bcl-2 protein | Cell cycle | EU678310 | | | | x | | | | | | |
| Beta-1,3-glucan binding protein | Immunity | CF369126 | | | | | x | | | | | |
| Betain-homocystein methyltransferase 2 | Metabolism | CB617563 | | | | | | | | x | | |
| Beta-transducin repeat-containing | Cell cycle | CD648487 | | | | | x | | | | | |
| Beta-tubulin cofactor D | Cytoskeleton | EE677703 | | | | | | | x | | | |
| Big defensin 3 | Immunity | BG624524 | | | | | | | | | | |
| Bindin F lectin | Immunity | CF369228 | | | | | | | | | x | |
| Block of proliferation protein/ribosome biogenesis | Cell cycle | HS513801 | | | | x | | | | | | |
| BMP type 1b receptor | Comms | HS513821 | | | | x | | | | | | |
| Bone morphogenic protein | Comms | CK172353 | | | x | | | | | | | |
| BTD domain containing 9 | Cell cycle | EH645251 | | | | | x | | | | | |
| BTF3a | Transcription | CX069131 | | | | | | | | | | x |
| C1q-tumor necrosis factor | Comms | GH612394 | x | | | | | | | | | |
| CAAX prenyl protease 1-like protein | Protein regulation | CB617436 | | | | | | | | x | | |

| Gene/Protein | Intracellular Process | Accession # | QX | *B. ost* | *V. spl* | HV 1 | *P. mar* | temp high | temp low | HC | pest/ herb | hypox |
|---|---|---|---|---|---|---|---|---|---|---|---|---|
| Calcium/calmodulin-dependent protein kinase IV | Comms | CB617508 | | | | | | | | x | | |
| Calmodulin | Comms | CX069134 | | | | x | x | x | x | | | x |
| Calmodulin binding protein/Striatin | Comms | CD650655 | | | | | x | | | | | |
| Calponin-like protein | Cytoskeleton | EE677705 | | | | | | x | | | | |
| Calreticulin | Stress | CX069289 | | | | | | | | | | x |
| CAP, adenylate cyclase-associated protein 1 | Cytoskeleton | CX069294 | | | | | | | | | | x |
| Carbonic anhydrase | Metabolism | CX069170 | | | | | | | | | | x |
| Carbonic anhydrase precursor | Metabolism | CD649975 | | | | | x | | | | | |
| Carboxypeptidase (serine) CPVL | Protein regulation | GH612419 | x | | | | | | | | | |
| Carboxypeptidase B | Protein regulation | CX069279 | | | | | | | | | | x |
| Carnitine O-acetyltransferase | Metabolism | HS513823 | | | | x | | | | | | |
| Cartilage specific proteoglycan (600 AA)/ Aggrecan core protein | ECM | EE677706 | | | | | | | x | | | |
| Cathepsin B | Protein regulation | HS513799 | | | | x | | | | | | |
| Cathepsin L | Protein regulation | BQ426622 | | | x | | x | | | x | x | x |
| Cathepsin Z | Protein regulation | DW713843 | | | | | | x | | | | |

| Gene/Protein | Intracellular Process | Accession # | QX | *B. ost* | *V. spl* | HV 1 | *P. mar* | temp high | temp low | HC | pest/ herb | hypox |
|---|---|---|---|---|---|---|---|---|---|---|---|---|
| Cationic amino acid transporter | Pumps | EH644863 | | | | | x | | | | | |
| CCR4-NOT transcription complex subunit 1 | Transcription | CK172341 | | | x | | | | | | | |
| CDP-diacylglycerol synthase | Metabolism | CB617500 | | | | | | | | x | | |
| Cellulase | Metabolism | CX069160 | | | | | | | | | | x |
| Centrin 2 | Cytoskeleton | EE677711 | | | | | | | x | | | |
| Ceruloplasmin/Ferroxidase | Other | BQ426657 | | | x | | | | | | | |
| Chaperonin containing TCP1, subunit 3 (gamma) | Stress | EE677714 | | | | | | | | | | |
| Chaperonin containing TCP1, subunit 7 isoform b isoform 1 | Stress | EE677715 | | | | | | x | | | | |
| Citrate synthase | Metabolism | EE677716 | | | | | | x | | | | |
| Coactosin-like protein | Cytoskeleton | CD526821 | | | | | x | | | | | |
| Coatomer protein complex β-subunit | Translation | CB617439 | | | | | | | | x | | |
| Collagen alpha | ECM | CB617493 | | | | | | | | x | | |
| Collagen alpha-3(VI) chain | ECM | EE677767 | | | | | | | x | | | x |
| Collectin-12 | Immunity | BG624594 | | | | | | x | | | | |
| COMM domain-containing protein 5 | Unknown | CB617516 | | | | | | | | x | x | |
| Complement C1q-like protein 3/Gliacolin | Immunity | CX069269 | | | | | | | | | | x |

| Gene/Protein | Intracellular Process | Accession # | QX | *B. ost* | *V. spl* | HV 1 | *P. mar* | temp high | temp low | HC | pest/ herb | hypox |
|---|---|---|---|---|---|---|---|---|---|---|---|---|
| Complement component C1q | Immunity | GH612251 | x | | | | | | | | | |
| Complement receptor-1 like protein | Immunity | CX069321 | | | | | | | | | | x |
| Conserved hypothetical protein | Unknown | CV132138 | | | | | x | | | | | |
| C-type lectin 1 | Immunity | CV088356 | | | | | x | x | | | | x |
| C-type mannose receptor 2 | Immunity | CV088207 | | | | | x | | | x | | |
| Cyclin B3 | Cell cycle | CD646472 | | | | | x | | | | | |
| Cyclin G1 | Cell cycle | EH649368 | | | | | | | | | | |
| Cyclin L1 | Cell cycle | HS513803 | | | | | x | | | | | |
| Cyclophilin | Pumps | CK172388 | | | | x | | | | | | |
| Cystatin B | Protein regulation | CX069133 | | | | | | | x | | | x |
| Cysteine proteinase 3 | Protein regulation | EE677707 | | | | | | | x | | | |
| Cytochrome b | Metabolism | JF744720 | | | | | | x | | | | x |
| Cytochrome b-c1 complex subunit 7 | Metabolism | EE677877 | | | | | | | x | | | |
| Cytochrome c oxidase assembly protein COX15 | Metabolism | EH643958 | | | | | x | | | | | |
| Cytochrome c oxidase assembly protein COX19 | Metabolism | EE677721 | | | | | | x | | | | |

| Gene/Protein | Intracellular Process | Accession # | QX | *B. ost* | *V. spl* | HV 1 | *P. mar* | temp high | temp low | HC | pest/ herb | hypox |
|---|---|---|---|---|---|---|---|---|---|---|---|---|
| Cytochrome c oxidase subunit 1 | Metabolism | JF744642 | x | x | x | x | x | x | x | | | x |
| Cytochrome c oxidase subunit 2 | Metabolism | GH612363 | x | | | | | | | | | x |
| Cytochrome c oxidase subunit 3 | Metabolism | JF744643 | x | | | | | | x | x | | x |
| Cytochrome c oxidase subunit VIA, putative | Metabolism | JF744721 | | x | | | | | | | | |
| Cytochrome c1 heme protein | Metabolism | JF744737 | | x | | | x | | | | | |
| Cytochrome P450 1A1 | Stress | CB617404 | | | | | | | | x | | |
| Cytochrome P450 3A29 | Stress | GH612330 | x | | | | | | | | | |
| Cytochrome P450 nifedipine oxidase (P450 3A4) | Stress | CB617459 | | | | | | | | x | | |
| Cytoplasmic dynein intermediate chain | Cytoskeleton | DW713877 | | | | | | | x | | | |
| Cytoplasmic intermediate filament protein A | Cytoskeleton | EE677724 | | | | | | | x | | | |
| Cytoskeleton-associated protein 2/CTCL tumor antigen se20-10 | Cytoskeleton | AF177227 | | | | | | x | | | | |
| Dehydrogenase with MaoC-like domain | Metabolism | DW713819 | | | | | | x | | | | |

| **Gene/Protein** | **Intracellular Process** | **Accession #** | **QX** | ***B. ost*** | ***V. spl*** | **HV 1** | ***P. mar*** | **temp high** | **temp low** | **HC** | **pest/ herb** | **hypox** |
|---|---|---|---|---|---|---|---|---|---|---|---|---|
| Deoxyribnuclease II beta | NAR | CD526732 | | | | | x | | | | | |
| Divalent cation tolerant protein CUTA | Metabolism | JF744644 | | x | | | | | | | | |
| DNA polymerase gamma | Cell cycle | EE677727 | | | | | | x | | | | |
| DNA replication licensing factor MCM5 | Cell cycle | CK172304 | | | x | | | | | | | |
| DNA topoisomerase I | Cell cycle | CX069118 | | | x | | | x | x | | | x |
| dolichyl-diphosphooligosaccharide--protein glycosyltransferase subunit STT3A-like [Aplysia californica] | Metabolism | EH649338 | | | | | x | | | | | |
| Dopamine-beta-hydroxylase | Metabolism | CX069193 | | | | | | | | | | x |
| Dual adaptor for phosphotyrosine and 3 phosphoinositides | Comms | JF744646 | | x | | | | | | | | |
| Dual oxidase maturation factor1 | Stress | GH612333 | x | | | | | | | | | |
| Dynein intermediate chain 1 | Cytoskeleton | EH647818 | | | | | x | | | | | |
| Dynein light chain | Cytoskeleton | JF744648 | | x | | | | | | | | |
| Dyp-type peroxidase | Stress | JF744696 | | x | | | | | | | | |
| Early meiotic induction protein 5, putative | Cell cycle | JF744722 | | x | | | | | | | | |
| ECSIT | Comms | BQ427193 | | | x | | | | | | | |

| Gene/Protein | Intracellular Process | Accession # | QX | *B. ost* | *V. spl* | HV 1 | *P. mar* | temp high | temp low | HC | pest/ herb | hypox |
|---|---|---|---|---|---|---|---|---|---|---|---|---|
| Ectonucleoside triphosphate diphosphohydrolase 1 | Metabolism | EH647492 | | | | | x | | | | | |
| Eef2-prov protein | Translation | CX069231 | | | | | | | | | | x |
| EF-hand domain containing 2 | Other | CD647305 | | | | | x | | | | | |
| elF-3 (eukaryotic Translation initiation factor 3 subunit 4) | Translation | JF744697 | | x | | | | | | | | |
| Elongation factor 1 alpha | Translation | CX069156 | x | | | x | x | x | x | x | | x |
| Elongation factor 1 delta | Translation | CX069125 | | | | | | | x | | | x |
| Elongation factor 2 | Translation | CB617558 | | | | | | | x | x | x | x |
| Elongator complex protein 1 | Transcription | DW713861 | | | | | | | x | | | |
| Endo alpha-1,4 polygalactosaminidase precursor | Metabolism | CX069284 | | | | | | | | | | x |
| Endo/excinuclease amino terminal domain, putative | Translation | DW713884 | | | | | | | x | | | |
| Endo-1,3-beta-d-glucanase | Metabolism | CV087718 | | | | | x | | | | | |
| Enolase-phosphatase E1 | Metabolism | GH612307 | x | | | | | | | | | |
| ENPP4 protein/ Ectonucleotide pyrophosphatase/phosphodiesterase family member 5 | NAR | CX069280 | | | | | | | | | | x |
| Eosinophil peroxidase | Stress | CB617557 | | | | | | | | x | | |
| Ependymin related protein-1 precursor | Other | CX069256 | | | | | | | | | | x |

| Gene/Protein | Intracellular Process | Accession # | QX | *B. ost* | *V. spl* | HV 1 | *P. mar* | temp high | temp low | HC | pest/ herb | hypox |
|---|---|---|---|---|---|---|---|---|---|---|---|---|
| Esophagael cancer associated protein (UPF0505 protein C16orf62) | Other | CX069352 | | | | | | | | | | x |
| Euchromatic histone-lysine N-methyltransferase 2 | Transcription | EH647103 | | | | | x | | | | | |
| Eukaryotic Translation elongation factor 6 | Translation | CX069250 | | | | | | | | | | x |
| Eukaryotic Translation initiation factor 3 subunit 6 | Translation | CX069268 | | | | | | | | | | x |
| Eukaryotic Translation initiation factor 3 subunit H | Translation | EE677730 | | | | | | | x | | | |
| Eukaryotic Translation initiation factor 4A isoform 1 | Translation | CX069326 | | | | | | | | | | x |
| Eukaryotic Translation initiation factor 6 | Translation | BG624116 | | | | | x | | | | | |
| Extracellular superoxide dismutase (Cu, Zn) | Stress | GH612244 | x | x | x | | | | x | x | x | x |
| F box protein FBL5 | Protein regulation | CX069124 | | | | | | | | | | x |
| Fas apoptotic inhibitory molecule | Cell cycle | EH644652 | | | | | x | | | | | |
| fasciclin | ECM | CB617561 | | | | | | | | x | | |
| Fascin | Cytoskeleton | EE677732 | | | | | | x | | | | |
| Fatty acid binding protein | Metabolism | CK172312 | | | x | | | | x | | | x |
| Fatty acyl-CoA reductase 1 | Metabolism | CX069303 | | | | | | x | | | | x |
| Fatty acyl-CoA reductase 1 | Metabolism | DW713858 | | | | | | x | | | | |

| **Gene/Protein** | **Intracellular Process** | **Accession #** | **QX** | ***B. ost*** | ***V. spl*** | **HV 1** | ***P. mar*** | **temp high** | **temp low** | **HC** | **pest/ herb** | **hypox** |
|---|---|---|---|---|---|---|---|---|---|---|---|---|
| F-box protein 8 | Protein regulation | EH646447 | | | | | x | | | | | |
| ferritin | Immunity | CB617552 | | | x | | | | | x | | |
| Fibrillin 3 | ECM | CX069292 | | | | | | | x | | | x |
| Fibrinogen c domain containing 1 | Immunity | EH648988 | | | | | x | | | | | |
| Fibrinogen/Fibroleukin | Immunity | GH612418 | x | | | | | | | | | |
| Fibropellin | ECM | CB617395 | | | | | | | | x | | |
| Fimbrin/plastin | Cytoskeleton | GH612282 | x | | | | | | | | | |
| FK506-binding protein | Translation | EH645600 | | | | | x | | | | | |
| Flavin adenine dinucleotide synthetase | Metabolism | HS513806 | | | | x | | | | | | |
| Flavin-containing monooxygenase 2 | Stress | GH612241 | x | | | | | | | x | | |
| Four jointed box 1 | Cell cycle | EE677735 | | | | | | | x | | | |
| Fructosamine-3-kinase relatedprotein | Metabolism | JF744698 | | x | | | | | | | | |
| FYVE and coiled-coil domain containing 1 | Cytoskeleton | DW713869 | | | | | | | x | | | |
| G protein a subunit q | Comms | CD526752 | | | | | x | | | | | |
| G protein-coupled receptor 48 | Comms | CB617400 | | | | | | | | x | | |
| Galectin 4 | Immunity | BQ426390 | | x | x | | | | | | | |
| Galectin 9 | Immunity | EE677739 | | | | | | x | | | | |

| Gene/Protein | Intracellular Process | Accession # | QX | *B. ost* | *V. spl* | HV 1 | *P. mar* | temp high | temp low | HC | pest/ herb | hypox |
|---|---|---|---|---|---|---|---|---|---|---|---|---|
| galectin 6 | Immunity | EH645010 | | | | | x | | | | | |
| Gamma hydroxybutyrate dehydrogenase | Metabolism | CD646623 | | | | | x | | | | | |
| Ganglioside GM2 activator precursor | Metabolism | CB617434 | | | | | | | | x | | |
| GAPDH glyceraldehyde-3-phosphate dehydrogenase | Metabolism | JF744656 | | x | | | | | | | | |
| Geminin | NAR | CB617390 | | | | | | | | x | | |
| General mitochondrial matrix processing protease 55 kDa subunit | Metabolism | EE677741 | | | | | | | x | | | |
| Ghitm-prov protein | Unknown | DW713838 | | | | | | x | | | | |
| Glutamate receptor 4 | Comms | EH644984 | | | | | x | | | | | |
| Glutamine synthetase | Metabolism | CX069169 | | | | | | x | | x | x | x |
| Glutathione S-transferase 3 | Stress | CD526720 | | | | | x | | | | | |
| Glutathione S-transferase omega class | Stress | CB617406 | | x | | | | | | x | | |
| Glutathione S-transferase P2 | Stress | CB617512 | | | | | | | | x | | x |
| Glycogen phosphorylase | Metabolism | CD526825 | | | | | x | | | | | x |
| Glyoxylate reductase/hydroxypyruvate reductase | Metabolism | DW713837 | x | | | | | x | | | | |
| Glypican | Cell cycle | EU678311 | | | | x | | | | | | |
| GTP binding protein alpha sub-unit | Comms | CD526737 | | | | | x | | | | | |

| **Gene/Protein** | **Intracellular Process** | **Accession #** | **QX** | ***B. ost*** | ***V. spl*** | **HV 1** | ***P. mar*** | **temp high** | **temp low** | **HC** | **pest/ herb** | **hypox** |
|---|---|---|---|---|---|---|---|---|---|---|---|---|
| GTPase cRac1A | Comms | GH612359 | x | | | | | | | | | |
| Guanine nucleotide binding protein G(s) subunit | Comms | CD526828 | | | | | x | | | | | |
| Guanine nucleotide exchange factor VAV2 | Comms | BQ427355 | | | x | | | | | | | |
| Guanine nucleotide-binding protein beta subunit-like protein | Comms | CX069147 | | | | | | | | | | x |
| Guanine nucleotide-binding protein G(1) alpha subunit | Comms | GH612348 | x | | | | | | | | | |
| Guanyl cyclase 32E | Comms | CF369176 | | | | | | | | | x | |
| H+ -transporting ATPase subunit d | Metabolism | CB617449 | | | | | | | | x | | |
| HAUS augmin-like complex subunit 8 | Cytoskeleton | EE677803 | | | | | | x | | | | |
| Heart-type fatty acid-binding protein | Metabolism | EE677746 | | | | | | | x | | | |
| Heat shock 70 kDa protein 12B | Stress | DW713816 | | | | | | x | | | | |
| Heat shock factor binding protein 1 | Stress | BG624136 | | | | | | | | | | |
| Heat shock protein 70 | Stress | CX069205 | | | | | | x | | x | | x |
| Heat shock transcription factor | Stress | CD526741 | | | | | x | | | | | |
| Hemagglutinin/amebocyte aggregation factor precursor | Immunity | EE677748 | | | | | | x | | | | |

| Gene/Protein | Intracellular Process | Accession # | QX | *B. ost* | *V. spl* | HV 1 | *P. mar* | temp high | temp low | HC | pest/ herb | hypox |
|---|---|---|---|---|---|---|---|---|---|---|---|---|
| Hemicentin-1 | ECM | DW713862 | | | x | | | | x | | | x |
| Hemocyte defensin Defh1 | Immunity | EE677749 | | | | | | | x | | | |
| Hepatic lectin | Immunity | CB617560 | | | | | | | | x | | |
| Heterogenous nuclear ribonucleoprotein AB isoform a | Transcription | HS513785 | | | | x | | | | | | |
| High mobility group 1 protein; HMG1 | Transcription | CX069137 | | | | | | | | | | x |
| Histone deacetylase complex subunit SAP18 | Transcription | EE677750 | | | | | | | x | | | |
| Histone H2b | Transcription | BG624428 | | | | | x | | | | | |
| Histone H3.3 | Transcription | BG624455 | | | | | x | | | | | x |
| Histone H4 | Transcription | DW713865 | | | | | | | x | | | |
| Homologue of ES1 | Unknown | CX069263 | | | | | | | | | | x |
| HSP 40 (DnaJ) homolog superfamily B 4 | Stress | JF744728 | | x | | | | | | | | |
| HSP90 | Stress | CB617443 | | x | | | | | | x | | |
| Huntingtin interacting protein K | Unknown | EE677751 | | | | | | | x | | | |
| IgGFc-binding protein | Immunity | GH612263 | x | | | | | | | | | |
| IK cytokine | Comms | GH612255 | x | | | x | | | | | | |
| IkappaB | Comms | GH612349 | x | | | | | | | | | |

| Gene/Protein | Intracellular Process | Accession # | QX | *B. ost* | *V. spl* | HV 1 | *P. mar* | temp high | temp low | HC | pest/ herb | hypox |
|---|---|---|---|---|---|---|---|---|---|---|---|---|
| Immunoglobulin domain cell adhesion molecule | ECM | EU678312 | | | | x | | | | | | |
| Importin beta-2 | Comms | HS513825 | | | | x | | | | | | |
| Importin α2 | Comms | CB617497 | | | | | | | | x | | |
| Inactive hydroxysteroid dehydrogenase-like protein 1 | Metabolism | EU108712 | | | | | | | | | | x |
| Inhibitor of apoptosis IAP 1 | Cell cycle | EH644983 | | | | | x | | | | | |
| Inhibitor protein Kappa B (of nuclear factor) | Comms | BQ427181 | | | x | | | | | | | |
| Inner membrane protein OXA1L | Metabolism | EE677759 | | | | | | x | | | | |
| Integrin | Comms | BQ426737 | | | x | | | | | | | |
| Integrin alpha 4 | Comms | EH645734 | | | | | x | | | | | |
| Interferon-induced protein 44 | Immunity | FJ440108 | | | | x | x | | | | | |
| Intersectin-1 | Cytoskeleton | CD526734 | | | | | x | | | | | |
| Intersectin-1 | Cytoskeleton | CD526818 | | | | | x | | | | | |
| IQ motif containing G | Comms | DW713870 | | | | | | | x | | | |
| Isocitrate dehydrogenase | Metabolism | CK172330 | | | x | | | | | | | |

| Gene/Protein | Intracellular Process | Accession # | QX | *B. ost* | *V. spl* | HV 1 | *P. mar* | temp high | temp low | HC | pest/ herb | hypox |
|---|---|---|---|---|---|---|---|---|---|---|---|---|
| IWS1-like protein | Transcription | GH612276 | x | | | | | | | | | |
| Krueppel-like factor 5 | Transcription | DW713853 | | | | | | x | | | | |
| Lanthionin synthetase C-like protein | Unknown | CD526726 | | | | | x | | | | | |
| L-ascorbate oxidase | Metabolism | CX069275 | | | | | | | | | | x |
| Latrophilin-3 [Crassostrea gigas] | Comms | CX069029 | | | | | | | | | | x |
| Legumain | Protein regulation | GH612370 | x | | | | | | | | | |
| Lipopolysaccharide and beta-1,3-glucan binding protein | Immunity | CX069184 | | | | | | | | x | | x |
| Lipoprotein receptor-related protein 5 | Comms | CB617399 | | | | | | | | x | x | |
| Low density lipoprotein receptor | Comms | EE677762 | | | | | | x | | | | |
| L-rhamnose-binding lectin CSL3 | Immunity | EE677761 | x | | | | | | x | | | |
| Lys-63-specific deubiquitinase BRCC36 | Protein regulation | CD526740 | | | | | x | | | | | |
| Lysosomal associated transmembrane protein | Pumps | CD526837 | | | | | x | | | | x | |
| Lysosomal-trafficking regulator | Cytoskeleton | CD526748 | | | | | x | | | | | |
| Lysozyme | Immunity | CB617495 | | | | | | | | x | | |
| Macrophage expressed protein 1 like protein | Unknown | EF627979 | | | | x | | | | | | |
| Malate dehydrogenase | Metabolism | CB617551 | | | | | | | | x | | |

| Gene/Protein | Intracellular Process | Accession # | QX | *B. ost* | *V. spl* | HV 1 | *P. mar* | temp high | temp low | HC | pest/ herb | hypox |
|---|---|---|---|---|---|---|---|---|---|---|---|---|
| Mammalian ependymin related protein 1 | Unknown | CB617379 | | | | | | | | x | | |
| MAPK 8 interacting protein/C-Jun-amino-terminal kinase-interacting protein 1 | Comms | CD526707 | | | | | x | | | | | |
| Matrix metalloproteinase | Protein regulation | GH612373 | x | | | | | | | | | |
| MEGF, Multiple epidermal growth factor-like domains 6 | Comms | HS513812 | | | | x | | | | | | |
| Meningioma expressed antigen 5/Bifunctional protein NCOAT | Transcription | CF369226 | | | | | | | | x | x | |
| Meprin A subunit beta | Unknown | CB617388 | | | | | | | | x | | |
| Mesoderm specific transcript | Unknown | HS513793 | | | | x | | | | | | |
| Metalloproteinase | Protein regulation | BQ426653 | | | x | | | | | | | |
| Metallothionein | Stress | CX069233 | x | x | x | | x | | | | | x |
| Metallothionein iie | Stress | CV133159 | | | | | x | | | | | |
| Metallothionein IV | Stress | CV087794 | | | | | x | | | | | |
| Metastasis associated 1 family, member 3 | Transcription | CD647013 | | | | | x | | | | | |
| Metastasis associated protein MTA1 | Transcription | DW713818 | | | | | | x | | | | |
| Methionine adenosyltransferase I, alpha | Metabolism | EE677769 | | | | | | x | | | | |

| Gene/Protein | Intracellular Process | Accession # | QX | *B. ost* | *V. spl* | HV 1 | *P. mar* | temp high | temp low | HC | pest/ herb | hypox |
|---|---|---|---|---|---|---|---|---|---|---|---|---|
| Methionine aminopeptidase 1D | Stress | GH612309 | x | | | | | | | | | |
| Methylmalonate semialdehyde dehydrogenase | Metabolism | EE677770 | | | | | | | x | | | |
| Microsomal Glutathione S transferase | Stress | JF744700 | | x | | | | | | | | |
| Mitochondrial acyl carrier protein 1 CG9160-PA, isoform A | Metabolism | EE677773 | | | | | | | x | | | |
| Mitochondrial ATP synthase alpha-subunit | Metabolism | EE677774 | | | | | | x | | | | |
| Mitochondrial ATP synthase F chain | Metabolism | JF744662 | | x | | | | x | | | | |
| Mitochondrial ATP-synthase γ-subunit - gamma | Metabolism | CB617389 | | | | | | | | x | | |
| Mitochondrial import receptor subunit TOM70 | Pumps | HS513779 | | | | x | | | | | | |
| Mitochondrial processing peptidase β chain | Metabolism | CB617453 | | | | | | | | x | | |
| Mitochondrial ribosomal protein L50 | Translation | GH612250 | x | | | | | | | | | |
| Mitochondrial-processing peptidase subunit beta | Protein regulation | EE677878 | | | | | | | x | | | |

| **Gene/Protein** | **Intracellular Process** | **Accession #** | **QX** | ***B. ost*** | ***V. spl*** | **HV 1** | ***P. mar*** | **temp high** | **temp low** | **HC** | **pest/ herb** | **hypox** |
|---|---|---|---|---|---|---|---|---|---|---|---|---|
| Mitotic apparatus protein p62 | Cell cycle | GH612252 | x | | | | | | | | | |
| Mitotic spindle assembly checkpoint protein MAD2B | Cell cycle | CV132655 | | | | | x | | | | | |
| Monoamine oxidase | Metabolism | CB617454 | | | | | | | | x | | |
| Mortality factor 4 like 1 | Transcription | GH612336 | x | | | | | | | | | |
| Multi EGF-like domains 6 | Cell cycle | HS513784 | | | | x | | | | | | |
| Multicopper oxidase | Stress | EU678320 | | | | x | | | | | | |
| MyD88 | Cell cycle | BQ426631 | | | x | | | | | | | |
| Myosin subunit essential light chain | Cytoskeleton | CX069307 | | | | | | x | | | | x |
| Myosin, heavy polypepetide 2 | Cytoskeleton | JF744729 | | x | | | | | | | | |
| Myosin-11 | Cytoskeleton | HS513797 | | | | x | | | | | | |
| N-acetyltransferase | Metabolism | CX069224 | x | | | | | | | | | x |
| NADH dehydrogenase (ubiquinoa) 1 β complex | Metabolism | JF744666 | | x | | | | | | | | |
| NADH dehydrogenase (ubiquinone) Fe-S protein | Metabolism | EE677777 | | | | | | | x | | | |
| NADH dehydrogenase [ubiquinone] iron-sulfur protein 2, mitochondrial | Metabolism | CD526722 | | | | | x | | | | | |

| Gene/Protein | Intracellular Process | Accession # | QX | *B. ost* | *V. spl* | HV 1 | *P. mar* | temp high | temp low | HC | pest/ herb | hypox |
|---|---|---|---|---|---|---|---|---|---|---|---|---|
| NADH dehydrogenase 6 | Metabolism | CK172316 | | x | x | | | | | | | x |
| NADH dehydrogenase flavoprotein 3 | Metabolism | CV088395 | | | | | x | | | | | |
| NADH dehydrogenase subunit 1 | Metabolism | JF744668 | | x | | | | x | x | x | | x |
| NADH dehydrogenase subunit 2 | Metabolism | JF744669 | | x | | | | x | x | | | |
| NADH dehydrogenase subunit 3 | Metabolism | JF744670 | | x | | | | | | x | | x |
| NADH dehydrogenase subunit 5 | Metabolism | JF744671 | | x | | x | x | x | | x | x | x |
| NADH-ubiquinone oxidoreductase 20kDa subunit, mitochondrial precursor | Metabolism | CB617510 | | | | | | | | x | | |
| NADH-ubiquinone oxidoreductase 49 kDa subunit | Metabolism | EE677778 | | | | | | x | | | | |
| NADPH oxidase 5 | Metabolism | HS513788 | | | | x | | | | | | |

| **Gene/Protein** | **Intracellular Process** | **Accession #** | **QX** | ***B. ost*** | ***V. spl*** | **HV 1** | ***P. mar*** | **temp high** | **temp low** | **HC** | **pest/ herb** | **hypox** |
|---|---|---|---|---|---|---|---|---|---|---|---|---|
| NASCENT polypeptide-associated complex α polypeptide | Translation | CB617385 | | | | | | x | | x | | |
| Neuroendocrine convertase 1 | Protein regulation | EE677804 | | | | | | | x | | | |
| Nicastrin | Protein regulation | EU678321 | | | | x | | | | | | |
| Non-selenium glutathione peroxidase | Stress | GH612366 | x | | | | | | x | | | x |
| Notch 3 | Comms | EF999949 | | | | x | | | | | | |
| Nuclear receptor subfamily 0 group B member 1 | Comms | GH612365 | x | | | | | | | | | |
| Nuclear RNA helicase | Transcription | CB617405 | | | | | | | | x | | |
| Nucleoporin/Nuclear pore complex Nup98-Nup96 | Translation | HS513786 | | | | x | | | | | | |
| Nucleoredoxin | Stress | BG624216 | | | | | | | | | | |
| Nucleoside-diphosphate kinase 2 | Metabolism | EE677783 | | | | | | x | | | | |
| Nucleotidase 4F8 | Comms | HS513826 | | | | x | | | | | | |
| O-6-Methylguanine-DNA methyltransferase | NAR | JF744730 | | x | | | | | | | | |
| Omega-amidase NIT2 | Stress | CB617446 | | | | | | | | x | | |
| Organic cation transporter protein | Pumps | EH647141 | | | | | x | | | | | |

| Gene/Protein | Intracellular Process | Accession # | QX | *B. ost* | *V. spl* | HV 1 | *P. mar* | temp high | temp low | HC | pest/ herb | hypox |
|---|---|---|---|---|---|---|---|---|---|---|---|---|
| Ornithine decarboxylase | Metabolism | GH612342 | x | | | | | | | | | |
| PAI-1 mRNA-binding protein/Plasminogen activator inhibitor 1 RNA-binding protein | Translation | EE677785 | | | | | | x | | | | |
| Patched domain of vertebrates | Unknown | JF744701 | | x | | | | | | | | |
| PDZ and LIM domain protein 5 | Protein regulation | CD526717 | | | | | x | | | | | |
| Pentraxin | Immunity | EE677736 | | | | | | | x | | | |
| Peptidase (mitochondrial processing) alpha | Metabolism | EE677788 | | | | | | | x | | | |
| Peptide methionine sulfoxide reductase | Stress | HS513802 | | | | x | | | | | | |
| Peripheral-type benzodiazepine receptor/translocator protein | Pumps | EE677789 | | | | | | x | | | | |
| Pernin precursor/dominin precursor | Stress | CD526735 | | | | | x | | | | | |
| Peroxiredoxin 6 | Stress | EH644464 | | | | | x | | | | | |
| peroxisome biogenesis factor | Stress | CX069207 | | | | | | | | | | x |
| Pescadillo homolog | Cell cycle | CD526714 | | | | | x | | | | | |
| PHD finger protein 10 | Transcription | EU678315 | | | | x | | | | | | |
| Phenylalanine hydroxylase | Metabolism | EE677790 | | | | | | | x | | | |

| Gene/Protein | Intracellular Process | Accession # | QX | *B. ost* | *V. spl* | HV 1 | *P. mar* | temp high | temp low | HC | pest/ herb | hypox |
|---|---|---|---|---|---|---|---|---|---|---|---|---|
| Phosphoenolpyruvate carboxykinase | Metabolism | CB617458 | | | | | x | | | x | | |
| Phosphoinositide 4-kinase beta | Comms | EU678316 | | | | x | | | | | | |
| Phospholipase C | Comms | EF627978 | | | | x | | | | | | |
| Placental protein 11 | Unknown | CD526820 | | | | | x | | | | | |
| Plasminogen | Protein regulation | HS513831 | | | | x | | | | | | |
| Poly (ADP-ribose) polymerases | Cell cycle | CB617394 | | | | | | | | x | | |
| Poly(a)-specific ribonuclease | Translation | CK172399 | | | x | | | | | | | |
| Polyamine N-acetyltransferase (spermidine) | Cell cycle | CX069230 | | | | | | | | | | x |
| polypirimidine tract binding 2 protein | Transcription | HS513817 | | | | x | | | | | | |
| Polypyrimidine tract binding protein 2 | Translation | JF744702 | | x | | | | | | | | |
| PP2A inhibitor/protein SET | Unknown | CK172303 | | | x | | | | | | | x |
| Predicted cation transporter | Pumps | DW713820 | | | | | | x | | | | |
| Prefolding 5 | Stress | JF744674 | | x | | | | | | | | |
| PRELI domain-containing protein 1, mitochondrial | Unknown | DW713831 | | | | | | x | | | | |
| Proactivator polypeptide | Protein regulation | CX069257 | | | | | | | | | | x |
| Probable histone-binding protein Caf1 | Cell cycle | CB617555 | | | | | | | | x | | |

| Gene/Protein | Intracellular Process | Accession # | QX | *B. ost* | *V. spl* | HV 1 | *P. mar* | temp high | temp low | HC | pest/ herb | hypox |
|---|---|---|---|---|---|---|---|---|---|---|---|---|
| Profilin | Cytoskeleton | EE677791 | | x | | | x | x | | | | |
| Prohormone convertase 1/Neuroendocrine convertase 1 | Comms | CX069297 | | | | | | | x | | | x |
| Prolactin regulatory element-binding protein | Transcription | EH644443 | | | | | x | | | | | |
| prostaglandin E synthase | Metabolism | CB617437 | | | | | | | | x | | |
| Proteasome iota chain | Protein regulation | CD526846 | | | | | x | | | | | |
| Proteasome maturation protein | Protein regulation | EE677827 | | | | | | x | | | | |
| Protein (receptor for activated protein kinase C) | Comms | DW713891 | | | | | | | x | | | |
| Protein disulfide-isomerase A6 precursor | Stress | CX069278 | | | | | | | | | | x |
| Protein lethal(2)essential for life | Unknown | CX069265 | | | | | | | | | | x |
| Protein lin-10 | Unknown | HS513809 | | | | x | | | | | | |
| Protein tyrosine phosphatase | Comms | EU678317 | | | | x | | | | | | |
| Protein unc-45 homolog A | Stress | HS513800 | | | | x | | | | | | |
| Proteo 26S non-ATPase subunit 1 | Protein regulation | CX069258 | | | | | | | | | | x |
| Putative 28kDa protein, partner of Nob1 | Unknown | CX069208 | | | | | | | | | | x |
| Putative 2-acylglycerol o-acyltransferase 1 | Metabolism | GH612386 | x | | | | | | | | | |

| Gene/Protein | Intracellular Process | Accession # | QX | *B. ost* | *V. spl* | HV 1 | *P. mar* | temp high | temp low | HC | pest/ herb | hypox |
|---|---|---|---|---|---|---|---|---|---|---|---|---|
| Putative alpha actinin-1 | Cytoskeleton | GH612297 | x | | | | | | | | | |
| Putative cation transport regulator-like protein 1 | Pumps | GH612408 | x | | | | | | | | | |
| Putative cysteine rich motor neuron2 protein/Kielin/chordin-like protein | Comms | GH612397 | x | | | | | | | | | |
| Putative Delta-aminolevulinic acid dehydratase | Metabolism | GH612322 | x | | | | | | | | | |
| Putative early growth response 1 | Cell cycle | GH612294 | x | | | | | | | | | |
| Putative enoyl-CoA hydratase/isomerase family/Fatty acid oxidation complex subunit alpha | Metabolism | CX069345 | | | | | | | | | | x |
| Putative eukaryotic Translation initiation factor 1b | Translation | GH612300 | x | | | | | | | | | |
| Putative G2/mitotic-specific cyclin-A | Cell cycle | GH612281 | x | | | | | | | | | |
| Putative HMG-like protein | Transcription | AJ563482.1 | | | | | | | | | | x |
| Putative proline-rich transmembrane protein | Pumps | GH612396 | x | | | | | | | | | |
| putative pyridoxine biosynthesis SNZERR | Metabolism | CX069189 | | | | | | | | | | x |

| Gene/Protein | Intracellular Process | Accession # | QX | *B. ost* | *V. spl* | HV 1 | *P. mar* | temp high | temp low | HC | pest/ herb | hypox |
|---|---|---|---|---|---|---|---|---|---|---|---|---|
| putative pyruvate dehydrogenase | Metabolism | GH612388 | x | | | | | | | | | |
| Putative RecQ family DNA helicase protein | NAR | GH612302 | x | | | | | | | | | |
| Putative SEC13 homolog | Translation | GH612304 | x | | | | | | | | | |
| Putative senescence-associated protein | Cell cycle | CF369134 | | | | | | | | | x | |
| Putative serine protease | Protein regulation | EH645736 | | | | | x | | | | | |
| Putative signal peptide CUB-EGF-like domain-containing protein | Comms | GH612286 | x | | | | | | | | | |
| Putative sphingosine-1-phosphate lyase | Metabolism | CX069242 | | | | | | | | | | x |
| QM protein | Stress | EE677795 | | | | | | | x | | | |
| QM-like protein | Stress | EE677794 | | | | | | | x | | | |
| Ra1A binding protein | Comms | CD526729 | | | | | x | | | | | |
| Ra1BP1 protein | Comms | CD526836 | | | | | x | | | | | |
| Rab acceptor 1 | Comms | EH647696 | | | | | x | | | | | |
| Rab GTPase-activating protein 1 | Comms | EE677796 | | | | | | x | | | | |
| Rab20-like protein | Comms | AF288677 | | | | | | | | x | | |
| RAB21 | Comms | CD526747 | | | | | x | | | | | |

| Gene/Protein | Intracellular Process | Accession # | QX | *B. ost* | *V. spl* | HV 1 | *P. mar* | temp high | temp low | HC | pest/ herb | hypox |
|---|---|---|---|---|---|---|---|---|---|---|---|---|
| RAB32 | Comms | CB617401 | | | | | | | | x | | |
| Rab5 | Comms | EE677797 | | | | | | x | | | | |
| Rab5 GDP/GTP exchange factor | Comms | CD526823 | | | | | x | | | | | |
| Rab9 effector | Comms | CD526824 | | | | | x | | | | | |
| RACK | Comms | DW713845 | | | | | | x | | | | |
| Ran protein | Comms | CX069126 | | | | | | | | | | x |
| Ras-like GTP-binding protein Rho1 | Comms | HS513782 | | | | x | | | | | | |
| Ras-related protein Rab-1A | Comms | HS513805 | | | | x | | | | | | |
| Ras-related protein Ral-A | Comms | HS513813 | | | | x | | | | | | |
| RHO GTPase | Comms | CD526744 | | | | | x | | | | | |
| Rho GTPase-activating protein | Comms | EH648693 | | | | | x | | | | | |
| Rho-GDI related protein | Comms | HS513816 | | | | x | | | | | | |
| Rho-related GTP-binding protein RhoE | Comms | EH648161 | | | | | x | | | | | |
| Rhotekin | Comms | EH645903 | | | | | x | | | | | |

| Gene/Protein | Intracellular Process | Accession # | QX | *B. ost* | *V. spl* | HV 1 | *P. mar* | temp high | temp low | HC | pest/ herb | hypox |
|---|---|---|---|---|---|---|---|---|---|---|---|---|
| Ribonuclease | NAR | GH612298 | x | | | | | | | | | |
| Ribonucleoside-diphosphate reductase (M2 like) | NAR | GH612242 | x | | | | | | | | | |
| Ribonucleotide reductase m1 | NAR | CD649253 | | | | | x | | | | | |
| Ribosomal protein 40S S2 | Translation | CF369248 | | | | | | | | | x | |
| Ribosomal protein 60S P2 | Translation | CF369252 | | | | | | | | | x | |
| Ribosomal protein L10 | Translation | DW713840 | | | | | | x | x | | | |
| Ribosomal protein L10a | Translation | CX069331 | | | | | | | x | | | |
| Ribosomal protein L12 | Translation | CX069140 | | x | | | | | x | | | |
| Ribosomal protein L14 | Translation | EE677832 | | | | | x | x | x | | x | |
| Ribosomal protein L17A | Translation | EE677835 | | | | | | x | x | | x | |
| Ribosomal protein L18 | Translation | AJ563457 | | | | | | x | x | | | |
| Ribosomal protein L18a | Translation | CF369129 | | | | | | x | x | | x | |
| Ribosomal protein L21 | Translation | EE677838 | | | | | | x | x | | | |
| Ribosomal protein L23a | Translation | EE677810 | | | | | | | x | | | |
| Ribosomal protein L24 | Translation | EE677813 | | | | | | | x | | | |
| Ribosomal protein L26 | Translation | EE677816 | | | | | | x | | | | |
| Ribosomal protein L27 | Translation | CF369246 | | | | | | x | | | x | |
| Ribosomal protein L3 | Translation | CB617375 | | | | | | x | | | | |
| Ribosomal protein L30 | Translation | DW713879 | | | | | | | x | | | |
| Ribosomal protein L34 | Translation | EE677819 | | | x | x | | | x | | | |
| Ribosomal protein L35 | Translation | CD526721 | | | | | x | | x | | | |

| Gene/Protein | Intracellular Process | Accession # | QX | *B. ost* | *V. spl* | HV 1 | *P. mar* | temp high | temp low | HC | pest/ herb | hypox |
|---|---|---|---|---|---|---|---|---|---|---|---|---|
| Ribosomal protein L35a | Translation | DW713886 | | | | | | | x | | | |
| Ribosomal protein L37 | Translation | CD526724 | | x | | | x | | | | | |
| Ribosomal protein L38 | Translation | CF369177 | | | | | | | | | x | |
| Ribosomal protein L39 | Translation | JF744704 | | x | | | | | | | | |
| Ribosomal protein L5 | Translation | CB617373 | | | | | | | x | | | |
| Ribosomal protein L8 (rp11 gene) | Translation | JF744678 | | x | | | | | x | | | |
| Ribosomal protein L9 | Translation | CB617372 | | | | | x | | x | | | |
| Ribosomal protein P2-like | Translation | DW713873 | | | | | | | x | | | |
| Ribosomal protein rp16 | Translation | GH612327 | x | | | | | | | | | |
| Ribosomal protein RSP14A | Translation | DW713881 | | | | | | | x | | | |
| Ribosomal protein S10 | Translation | AJ561117 | | | | | | | x | | | |
| Ribosomal protein S11 | Translation | AJ563454 | | | | | | x | x | | x | |
| Ribosomal protein S13 | Translation | EE677829 | | | | | | | x | | | |
| Ribosomal protein S14 | Translation | EE677833 | | | | | x | x | x | | | |
| Ribosomal protein S15 | Translation | EE677836 | | | | | | | | | | |
| ribosomal protein S15a | Translation | CK172301 | | | x | | | | | | | |
| Ribosomal protein S16 | Translation | CB617376 | | | | | | | x | | | |
| Ribosomal protein S18 | Translation | CF369145 | | | | | | | | | x | |
| Ribosomal protein S19 | Translation | GH612321 | x | | | | | | | | | |
| Ribosomal protein S20 | Translation | CD526844 | | | | | x | | | | | |
| Ribosomal protein S23 | Translation | CD526842 | | | | | x | | | | | |

| Gene/Protein | Intracellular Process | Accession # | QX | *B. ost* | *V. spl* | HV 1 | *P. mar* | temp high | temp low | HC | pest/ herb | hypox |
|---|---|---|---|---|---|---|---|---|---|---|---|---|
| Ribosomal protein S25 | Translation | CF369224 | | | | | | | | | x | |
| Ribosomal protein S26 | Translation | JF744681 | | x | | | | | | | | |
| Ribosomal protein S27-1 | Translation | AJ563471 | | | | | | x | | | | |
| Ribosomal protein S30 | Translation | CD526709 | | | | | x | | | | | |
| Ribosomal protein S3a | Translation | CB617371 | | | | | | x | | | x | |
| Ribosomal protein S3B | Translation | CF369251 | | | | | | | | | x | |
| Ribosomal protein S4 | Translation | DW713829 | | | | | | x | x | | | |
| Ribosomal protein S5 | Translation | DW713844 | | | | | | x | | | x | |
| Ribosomal protein S6 | Translation | DW713815 | | | | | | x | | | x | |
| Ribosomal protein S8 | Translation | DW713821 | | | | | | x | x | | x | |
| Ring finger 10 protein | Transcription | CD526710 | | | | | x | | | | | |
| RNA Adenosin deaminase | NAR | CB617393 | | | | | | | | x | | |
| RNA helicase | NAR | CF369125 | | | | | | | | | x | |
| RNA polymerase B transcription factor 3/BTF3 | Transcription | GH612385 | x | | | | | | | | | |
| RNA polymerase III 53kDa subunit RPC4 | Transcription | CX069158 | | | | | | | | | | x |
| RNA-binding protein | Translation | JF744682 | | x | | | | | | | | |
| Scalloped-like transcription factor | Transcription | EH646343 | | | | | x | | | | | |
| S-crystallin SL11 | Unknown | EE677744 | | | | | | x | | | | |

| Gene/Protein | Intracellular Process | Accession # | QX | *B. ost* | *V. spl* | HV 1 | *P. mar* | temp high | temp low | HC | pest/ herb | hypox |
|---|---|---|---|---|---|---|---|---|---|---|---|---|
| Sdhb-prov protein | Metabolism | CX069267 | | | | | | | | | | x |
| SEC14-like 1 (S cerevisiae) | other | CD648418 | | | | | x | | | | | |
| Secretin receptor | Comms | EE677786 | | | | | | | x | | | |
| Semaphorin 2A precursor | other | EU678318 | | | | x | | | | | | |
| Serine/threonine-protein kinase 38 | Comms | EU678314 | | | | x | | | | | | |
| set1/Ash2 histone methyltransferase complex subunit ASH2-like | Metabolism | EH646453 | | | | | x | | | | | |
| Severin | Cytoskeleton | EE677740 | | | | | | | x | | | |
| Signal peptidase 25 kDA subunit | Translation | EE677806 | | | | | | | x | | | |
| Similar to anaphase promoting complex subunit 10 | Cell cycle | JF744725 | | x | | | | | | | | |
| Similar to C1q-related factor precursor | Immunity | CB617506 | | | | | | | | x | | |
| Similar to CDC-like kinase 2/Dual specificity protein kinase CLK2 | Cell cycle | CB617496 | | | | | | | | x | | |
| Small RHO1 GTPase | Comms | CD526835 | | | | | x | | | | | |
| SMT3 or SUMO (small ubiquitin related modifier) | Stress | JF744705 | | x | | | | | | | | |
| SnF7 | Translation | EU678319 | | | | x | | | | | | |

| **Gene/Protein** | **Intracellular Process** | **Accession #** | **QX** | ***B. ost*** | ***V. spl*** | **HV 1** | ***P. mar*** | **temp high** | **temp low** | **HC** | **pest/ herb** | **hypox** |
|---|---|---|---|---|---|---|---|---|---|---|---|---|
| Sodium- and chloride-dependent GABA transporter 2 | Pumps | CK172352 | | | x | | | | | | | |
| Sodium glucose cotransporter | Pumps | CK172416 | | | x | | | | | | | |
| Sodium-coupled monocarboxylate transporter 1 | Pumps | CX069249 | | | | | | | | | | x |
| Sodium-dependent glucose transporter 1 | Pumps | EH649185 | | | | | x | | | | | |
| Sodium-dependent neutral amino acid transporter B(0)AT3 [Crassostrea gigas] | Pumps | CB617382 | | | | | | | | x | | |
| Solute carrier 6 | Pumps | JF744706 | | x | | | | | | | | |
| Solute carrier family 23 member 2 | Pumps | CX069171 | | | | | | | | | | x |
| Solute carrier family 25 member 38 | Pumps | CB617452 | | | | | | | | x | | |
| Solute carrier family 30 member 6 | Pumps | EH646093 | | | | | x | | | | | |
| Solute carrier family 37 member 2 | Pumps | EH648237 | | | | | x | | | | | |
| Solute carrier family 39 (zinc transporter)/zip14 | Pumps | DW713824 | | | | | | x | | | | |
| Solute carrier family 6 member 5 | Pumps | CD649250 | | | | | x | | | | | |
| Spectrin alpha chain | Cytoskeleton | EE677811 | | | | | | x | | | | |

| Gene/Protein | Intracellular Process | Accession # | QX | *B. ost* | *V. spl* | HV 1 | *P. mar* | temp high | temp low | HC | pest/ herb | hypox |
|---|---|---|---|---|---|---|---|---|---|---|---|---|
| Spermatogenesis associated 6 | Unknown | CD648429 | | | | | x | | | | | |
| Spermidine synthase | Metabolism | CX069283 | | | | | | | | | | x |
| Spinster homolog 1 | Cell cycle | EH648330 | | | | | x | | | | | |
| Splicing factor 3B subunit 3 | Transcription | HS513792 | | | | x | | | | | | |
| Splicing factor U2AF | Transcription | CD526826 | | | | | x | | x | x | | |
| Splicing factor, arginine/serine-rich 8 isoform 1 | Transcription | HS513830 | | | | x | | | | | | |
| SRY-box containing gene 4a | Cell cycle | EH644916 | | | | | x | | | | | |
| Steroid 17-alpha-hydroxylase/17,20 lyase (CYP17a1) / Cyto P450 17a1 | Stress | CX069165 | | | | | | | | | | x |
| Sterol regulatory element binding protein 1 | Transcription | DW713854 | | | | | | x | | | | |
| Steryl-sulfatase precursor | Metabolism | EE677814 | | | | | | x | | | | |
| Stress-associated endoplasmic reticulum protein 2 | Stress | EE677839 | | | | | | x | | | | |
| Succinate dehydrogenase Ip subunit | Metabolism | DW713882 | | | | | | | x | | | |
| Sulfite oxidase | Metabolism | CB617450 | | | | | | | | x | | |
| Sulfotransferase 1C1 | Metabolism | CB617550 | | | | | | | | x | | |
| Suppressor of profilin/p41 of actin-related complex 2/3 | Cytoskeleton | EE677821 | | | | | | | x | | | |

| Gene/Protein | Intracellular Process | Accession # | QX | *B. ost* | *V. spl* | HV 1 | *P. mar* | temp high | temp low | HC | pest/ herb | hypox |
|---|---|---|---|---|---|---|---|---|---|---|---|---|
| SWI/SNF complex 170 kDa subunit | Transcription | CB617547 | | | | | | | | x | | |
| Syntaxin-binding protein 5 | Cytoskeleton | HS513794 | | | | x | | | | | | |
| T-cell activiation protein phosphatase 2C | Comms | CX069356 | | | | | | | | | | x |
| T-complex protein 1 subunit zeta | Translation | CB617445 | | | | | | | | x | | |
| T-complex protein1, delta subunit | Stress | GH612315 | x | | | | | | | | | |
| Tenascin-R/Restrictin | ECM | CB617514 | | | | | | x | | x | | x |
| Tetraspanin 27/CD82 antigen | Unknown | CD526719 | | | | | x | | | | | |
| Tetraspanin-33 | Unknown | CB617502 | | | | | | | | x | | |
| TGF beta-inducible nuclear protein 1 | Unknown | CX069355 | | | | | | | | | | x |
| Thioredoxin | Stress | JF744692 | | x | | | | | | | | |
| Thioredoxin domain containing 14 | Stress | JF744693 | | x | | | | | | | | |
| Threonine 3-dehydrogenase | Metabolism | CX069187 | | | | | | | | | | x |
| Tissue inhibitor of metalloproteinase | Protein regulation | BQ427105 | | x | x | | | | | | | |
| TM2 domain-containing protein 3 | Unknown | CB617559 | | | | | | | | x | | |
| TNF-alpha factor | Comms | EE677849 | | | | | | | x | | | |
| Toll-like receptor | Immunity | CD526746 | | | | | x | | | | | |

| Gene/Protein | Intracellular Process | Accession # | QX | *B. ost* | *V. spl* | HV 1 | *P. mar* | temp high | temp low | HC | pest/ herb | hypox |
|---|---|---|---|---|---|---|---|---|---|---|---|---|
| Transcription factor iib | Transcription | BG624447 | | | | | x | | | | | |
| Transcription factor Rel 2 | Transcription | HS513832 | | | | x | | | | | | |
| Transcription factor TF IIF subunit 2 | Transcription | EE677857 | | | | | | x | | | | |
| Transcription initiation factor IID | Transcription | GH612247 | x | | | | | | | | | |
| Transforming growth factor-beta-induced protein ig-h3 | ECM | CB617562 | | | | | | | | x | | |
| Transglutaminase/annulin | Metabolism | EE677859 | | | x | | | | x | | | |
| Transketolase 2-like | Metabolism | HS513811 | | | | x | x | | | | | |
| Translation initiation factor 4A-lll-like | Translation | HS513783 | | | | x | | | | | | |
| Translation initiation factor eIF-2B delta subunit | Translation | EE677861 | | | | | | | x | | | |
| Translation initiation factor SUI1 | Translation | EE677863 | | | | | | | x | | | |
| Translationally controlled tumour protein | Unknown | GH612265 | x | | | | | | | | | |
| Translocator protein | Pumps | CK240404 | | | | | x | | | | | |
| Translocon-associated protein, gamma subunit | Translation | HS513810 | | | | x | | | | | | x |
| Transmembrane protein 184C | Unknown | HS513798 | | | | x | | | | | | |
| Transmembrane protein 77 | Unknown | EH647902 | | | | | x | | | | | |
| Transmembrane protein HEM-2 | Unknown | CB617503 | | | | | | | | x | | |

| **Gene/Protein** | **Intracellular Process** | **Accession #** | **QX** | ***B. ost*** | ***V. spl*** | **HV 1** | ***P. mar*** | **temp high** | **temp low** | **HC** | **pest/ herb** | **hypox** |
|---|---|---|---|---|---|---|---|---|---|---|---|---|
| Transmembrane receptor (putative melatonin receptor) | Comms | HM034838 | | | | x | | | | | | |
| Triacylglycerol lipase | Metabolism | EE677866 | | | | | | x | | x | | |
| Tripartite motif protein 2 | Immunity | EE677868 | | | | | | | x | | | |
| Tripartite motif protein 45 | Immunity | CF369175 | | | | | | | | | x | |
| Tripartite motif-containing protein 45 | Transcription | CD526733 | | | | | x | | | | | |
| tRNA splicing 2' phosphotransferase | Translation | CK172320 | | | x | | | | | | | |
| Troponin c | Cytoskeleton | EH646757 | | | | | x | | | | | |
| Trypsin | Protein regulation | CB617494 | | | | | | | | x | | |
| Tubulin alpha | cytoskeleton | EE677872 | | | | | | x | x | | x | x |
| Tubulin beta | cytoskeleton | CF369141 | x | | | | | x | x | x | x | x |
| Tubulin, beta 2A variant | cytoskeleton | EE677873 | | | | | | | x | | | |
| Tumour suppressor candidate 3 | Cell cycle | GH612295 | x | | | | | | | | | |
| Tyrosine-protein kinase receptor Tie-1 | Comms | CD526723 | | | | | x | | | | | |
| UbiE/COQ5 methyltransferase / 3-demethylubiquinone-9 3-methyltransferase | Metabolism | EE677875 | | | | | | | x | | | |
| Ubiquitin | Protein regulation | CX069287 | | | | | | | x | | | x |

| **Gene/Protein** | **Intracellular Process** | **Accession #** | **QX** | ***B. ost*** | ***V. spl*** | **HV 1** | ***P. mar*** | **temp high** | **temp low** | **HC** | **pest/ herb** | **hypox** |
|---|---|---|---|---|---|---|---|---|---|---|---|---|
| Ubiquitin activating enzyme | Protein regulation | CB617402 | | | | | | | | x | | |
| Ubiquitin conjugating enzyme | Protein regulation | CX069212 | | | | | x | | | | | x |
| Ubiquitin conjugating enzyme E2 G2 | Protein regulation | JF744694 | | x | | | | | | | | |
| Ubiquitin fusion degradation 1-like | Protein regulation | EE677812 | | | | | | | x | | | |
| Ubiquitin specific peptidase 16/Ubiquitin carboxyl-terminal hydrolase 16 | Protein regulation | HS513829 | | | | x | | | | | | |
| Ubiquitin/ribosomal L40 fusion protein | Protein regulation | CX069286 | | | | | | | | | | x |
| Ubiquitin/ribosomal protein S27a fusion protein | Protein regulation | EE677815 | | | | | | | x | | | |
| Ubiquitin-conjugating enzyme E2W | Protein regulation | EH646812 | | | | | x | | | | | |
| Universal Stress protein UspA/ Stress response protein nhaX | Stress | DW713874 | | | | | | | x | | | |
| Vacuolar protein sorting 33B | Translation | EE677818 | | | | | | x | | | | |
| Vacuolar sorting protein vps29 | Translation | JF744707 | | x | | | | | | | | |
| Vitellogenin | Metabolism | CX069172 | | | | | | | | | | x |
| Voltage dependent anion selective channel protein 2 | Pumps | CX069174 | | | | x | | x | x | | | x |
| Wurst-like protein | Cytoskeleton | HS513815 | | | | x | | | | | | |

| **Gene/Protein** | **Intracellular Process** | **Accession #** | **QX** | ***B. ost*** | ***V. spl*** | **HV 1** | ***P. mar*** | **temp high** | **temp low** | **HC** | **pest/ herb** | **hypox** |
|---|---|---|---|---|---|---|---|---|---|---|---|---|
| Y-box fector homologue (APY1) | Transcription | CX069347 | | | | | | | | | | x |
| Yeast ino80-like protein | Transcription | CD647385 | | | | | x | | | | | |
| Zinc finger CCCH domain-containing protein 3 | Unknown | GH612291 | x | | | | | | | | | |
| Zinc finger homeodomain 3 | Unknown | EH644704 | | | | | x | | | | | |
| Zinc finger protein Noc | Cell cycle | EE677808 | | | | | | | x | | | |
| α2-macroglobulin domain-containing protein 6/Pregnancy zone protein | Protein regulation | BQ426790 | | | x | | | | | x | | |
| β-1,3-Glucan binding protein | Immunity | CF369126 | | | | | | | | x | x | |
| β-N-acetylhexosaminidase/Beta-hexosaminidase | Metabolism | CB617435 | | | | | | | | x | | |
